# Identification of *DAPK1* Promoter Hypermethylation as a Biomarker for Intra-Epithelial Lesion and Cervical Cancer: A Meta-Analysis of Published Studies, TCGA, and GEO Datasets

**DOI:** 10.3389/fgene.2018.00258

**Published:** 2018-07-17

**Authors:** Xue-bin Wang, Ning-hua Cui, Xia-nan Liu, Jun-fen Ma, Qing-hua Zhu, Shu-ren Guo, Jun-wei Zhao, Liang Ming

**Affiliations:** ^1^Department of Clinical Laboratory, The First Affiliated Hospital of Zhengzhou University, Zhengzhou, China; ^2^Department of Clinical Laboratory, Children's Hospital Affiliated to Zhengzhou University, Henan Children's Hospital, Zhengzhou Children's Hospital, Zhengzhou, China

**Keywords:** *DAPK1* promoter hypermethylation, cervical cancer, intra-epithelial lesion, TCGA database, GEO database, meta-analysis

## Abstract

**Background:** Promoter hypermethylation in death-associated protein kinase 1 (*DAPK1*) gene has been long linked to cervical neoplasia, but the established results remained controversial. Here, we performed a meta-analysis to assess the associations of *DAPK1* promoter hypermethylation with low-grade intra-epithelial lesion (HSIL), high-grade intra-epithelial lesion (HSIL), cervical cancer (CC), and clinicopathological features of CC.

**Methods:** Published studies with qualitative methylation data were initially searched from PubMed, Web of Science, EMBASE, and China National Knowledge Infrastructure databases (up to March 2018). Then, quantitative methylation datasets, retrieved from the Cancer Genome Atlas (TCGA) and Gene Expression Omnibus (GEO) databases, were pooled to validate the results of published studies.

**Results:** In a meta-analysis of 37 published studies, DAPK1 promoter hypermethylation progressively increased the risk of LSIL by 2.41-fold (*P* = 0.012), HSIL by 7.62-fold (*P* < 0.001), and CC by 23.17-fold (*P* < 0.001). Summary receiver operating characteristic curves suggested a potential diagnostic value of DAPK1 promoter hypermethylation in CC, with a large area-under-the-curve of 0.83, a high specificity of 97%, and a moderate sensitivity of 59%. There were significant impacts of DAPK1 promoter hypermethylation on histological type (odds ratio (OR) = 3.53, *P* < 0.001) and FIGO stage of CC (OR = 2.15, *P* = 0.003). Then, a pooled analysis of nine TCGA and GEO datasets, covering 13 CPG sites within DAPK1 promoter, identified eight CC-associated sites, six sites with diagnostic values for CC (pooled specificities: 74–90%; pooled sensitivities: 70–81%), nine loci associated with the histological type of CC, and all 13 loci with down-regulated effects on *DAPK1* mRNA expression.

**Conclusion:** The meta-analysis suggests that *DAPK1* promoter hypermethylation is significantly associated with the disease severity of cervical neoplasia. *DAPK1* methylation detection exhibits a promising ability to discriminate CC from cancer-free controls.

## Introduction

Cervical cancer (CC), the second most common gynecologic cancer worldwide (Torre et al., 2015), is characterized as a progressive process from low-grade squamous intra-epithelial lesion (LSIL) to high grade squamous intra-epithelial lesion (HSIL) and eventually to invasive carcinoma (Vale et al., 2013). Although infection with human papilloma virus (HPV) is casually linked to cervical neoplasia, most HPV-induced lesions are spontaneously regressed and do not progress to invasive cancer (Guan et al., 2012), suggesting the existence of other molecular changes involved in cancer progression.

DNA hypermethylation, occurred at CPG islands within the proximal promoter of tumor suppressor genes (TSGs), is a common epigenetic feature of cervical carcinoma, leading to the silencing of TSGs and carcinogenesis (Wentzensen et al., 2009). Death-associated protein kinase 1 (*DAPK1*) gene, a pro-apoptotic TSG, encodes an activator of a p19ARF/p53-dependent apoptotic checkpoint (Martoriati et al., 2005), whose expression is frequently lost in cancer cells as a result of promoter hypermethylation (Raveh et al., 2001). In 2001, Dong et al. first reported a significant association of *DAPK1* promoter hypermethylation with the risk and histological type of CC (Dong et al., 2001). Then, along with the increasing number of studies for *DAPK1* promoter hypermethylation and CC, two meta-analyses, pooling the data of 15 and 20 studies, respectively, consistently implied a positive correlation between *DAPK1* methylation status and CC (Xiong et al., 2014; Agodi et al., 2015). However, there was still no comprehensive review that systematically appraised the role of *DAPK1* promoter hypermethylation in LSIL, HSIL, and clinicopathological features of CC. Moreover, quantitative methylation data of DAPK1 from the Cancer Genome Atlas (TCGA) and Gene Expression Omnibus (GEO) databases were not investigated.

Thus, in this updated meta-analysis, by combining the data of 37 published studies, we first evaluated the effects of DAPK1 promoter hypermethylation on LSIL, HSIL, CC, and clinicopathological features of CC. Then, nine quantitative methylation datasets from TCGA and GEO databases were pooled to validate the results of published studies, and further analyze the associations of DAPK1 methylation levels with DAPK1 mRNA expression and diagnosis of CC.

## Materials and methods

### Literature search, eligibility criterion, and data extraction for published studies

This meta-analysis followed the recommendations of the PRISMA Statement (Moher et al., 2009). The literature search was conducted in PubMed, Web of Science, EMBASE, and China National Knowledge Infrastructure (CNKI) databases through March 2018 by using the combinations of the following keywords: (*DAPK1* or *DAPK-1* or *DAPK*) and (methylation or hypermethylation or promoter hypermethylation) and (cervical cancer/cervical carcinoma/cervical neoplasia or SIL/LSIL/HSIL or cervical intraepithelial neoplasia (CIN)/carcinoma *in situ* (CIS)/cervical dysplasia). References in retrieved articles and relevant reviews were also screened for potential studies.

Eligible studies should meet the following criterion: (1) observational studies using cohort, case-control, or case-only designs; (2) application of standard cervical biopsy or PAP smear cytology for the diagnosis of cervical neoplasia; (3) studies investigating the effects of *DAPK1* promoter hypermethylation on LSIL, HSIL, CC, or clinicopathological features of CC; (4) Studies providing the numbers or frequencies of *DAPK1* promoter hypermethylation for calculation of odds ratios (ORs) and their 95% confidence intervals (CIs); (5) written in English or Chinese. For articles with repeated data, only the largest or the most recent studies were included. Articles were excluded if they were case reports, abstracts, *in vitro* or pharmacological experiments, research for normal cervix or benign cervical diseases, and studies with incomplete data.

The following data were extracted from eligible studies by two independent reviewers (NC and XL): the first author's name, publication year, country and ethnicity, study design, sample size, source of controls, methods of methylation analysis, primer sets (Table S1), clinicopathological features, and study quality. Any discrepancy between two reviewers was resolved by consensus.

### Data extraction and analysis of TCGA and GEO datasets

First, we downloaded genome-wide methylation profiles of 307 CC tissues and three normal tissues from the TCGA CESC project (https://cancergenome.nih.gov/). Then, eight methylation microarray datasets, including GSE99511, GSE68339, GSE46306, GSE41384, GSE37020, GSE36637, GSE30760, and GSE20080, were collected from the GEO database (https://www.ncbi.nlm.nih.gov/gds) by using the following keywords: “Homo sapiens”, “Cervical cancer,” and “Methyation.” All datasets above used the Illumina HumanMethylation 450 or 27 K Beadchip to detect methylation signals. Methylation data of each dataset were separately normalized by a Beta Mixture Quantile dilation (BMIQ) strategy implemented in the R package, which had an advantage of correcting for different distributions of methylation signals between Infinium I and Infinium II probes (Teschendorff et al., 2013). Methylation levels at each CPG site were expressed as a β-value, which represented a ratio of the quantile-normalized methylation intensity to total locus intensity (methylation + unmethylation). For quality control, probes were excluded if they (1) had a low bead count of <3 in at least 5% of samples, (2) showed a detection-*P* > 0.05 in at least 5% of samples, or (3) contained genetic variants at or within 10 bp from the target CPG sites (Verlaat et al., 2018). As a result, 13 CPG sites in the *DAPK1* promoter region, located on the CPG islands investigated by published literatures, were selected as the object of this meta-analysis. Considering that the methylation data were extracted from the Illumina 450/27 K microarrays (including up to 5 × 10^5^ probes), we used a genome-wide significance threshold of *P* < 10^−7^ (Bonferroni corrected) in meta-analyses of these 13 CPG sites (Joubert et al., 2014).

### Quality assessment for included studies

Quality assessment for eligible studies was performed by two independent reviewers (SG and QZ) using a predefined scale modified from the REMARK (Altman et al., 2012) and BRISQ guidelines (Moore et al., 2011). As quality components, 18 items were considered, evaluating the scientific design, biospecimen management, methylation detection, confounder record, and statistical analysis of included studies (Table S2). Studies reporting more than 11 items were rated as high-quality.

### Statistical analyses

For qualitative methylation data from published literatures, ORs and their 95% CIs were estimated to assess the effects of *DAPK1* promoter hypermethylation on LSIL, HSIL, CC, and clinicopathological features of CC. For quantitative methylation data from TCGA and GEO databases, we calculated the standardized mean differences (SMDs) in methylation levels of CPG sites between cases and controls. The diagnostic value of qualitative and quantitative methylation data in CC was evaluated by a summary receiver operating characteristic (SROC) curve, which showed the sensitive, specificity, and area under the curve (AUC) of included studies.

Heterogeneity between studies was assessed by the Cochran's Q test and *I*^2^ statistic. *I*^2^ values larger than 25, 50, and 75% indicated low, moderate, and high heterogeneity, respectively (Higgins et al., 2003). If significant heterogeneity was observed (*P*_Q−test_ ≤ 0.1 or *I*^2^ ≥ 50%), overall effects were weighted using a random-effects model with the inverse variance method; otherwise, a fixed-effects model was used (Lu et al., 2014). To identify the possible source of heterogeneity, subgroup and meta-regression analyses were conducted, according to ethnicity, study quality, source of controls, and primer sets. Galbraith plots were also used to depict the influence of individual studies on overall heterogeneity (Pabalan et al., 2017). To validate the stability of pooling results, sensitivity analyses were carried out by sequential removal of individual studies or by omitting the contributors of heterogeneity spotted by Galbraith plots (Lu et al., 2014). Publication bias was appraised by visual inspection of funnel plots and by performing the Egger's test (Egger et al., 1997).

In the TCGA CESC dataset, the association between CPG sites of *DAPK1* and histological data of CC was assessed by the Mann–Whitney U test; the prognosis of CPG sites in CC was appraised by the Cox regression approach for overall (OS) and disease-free survival (DFS) analyses. Methylation quantitative trait locus (meQTL) analyses for *DAPK1* were tested by the Spearman correlation test. All statistical analyses were conducted with STATA 12.0 (StataCorp, College Station, TX, USA) and RevMan 5.2 programs (The Cochrane Collaboration).

## Results

### Study characteristics

**Figure 1:**
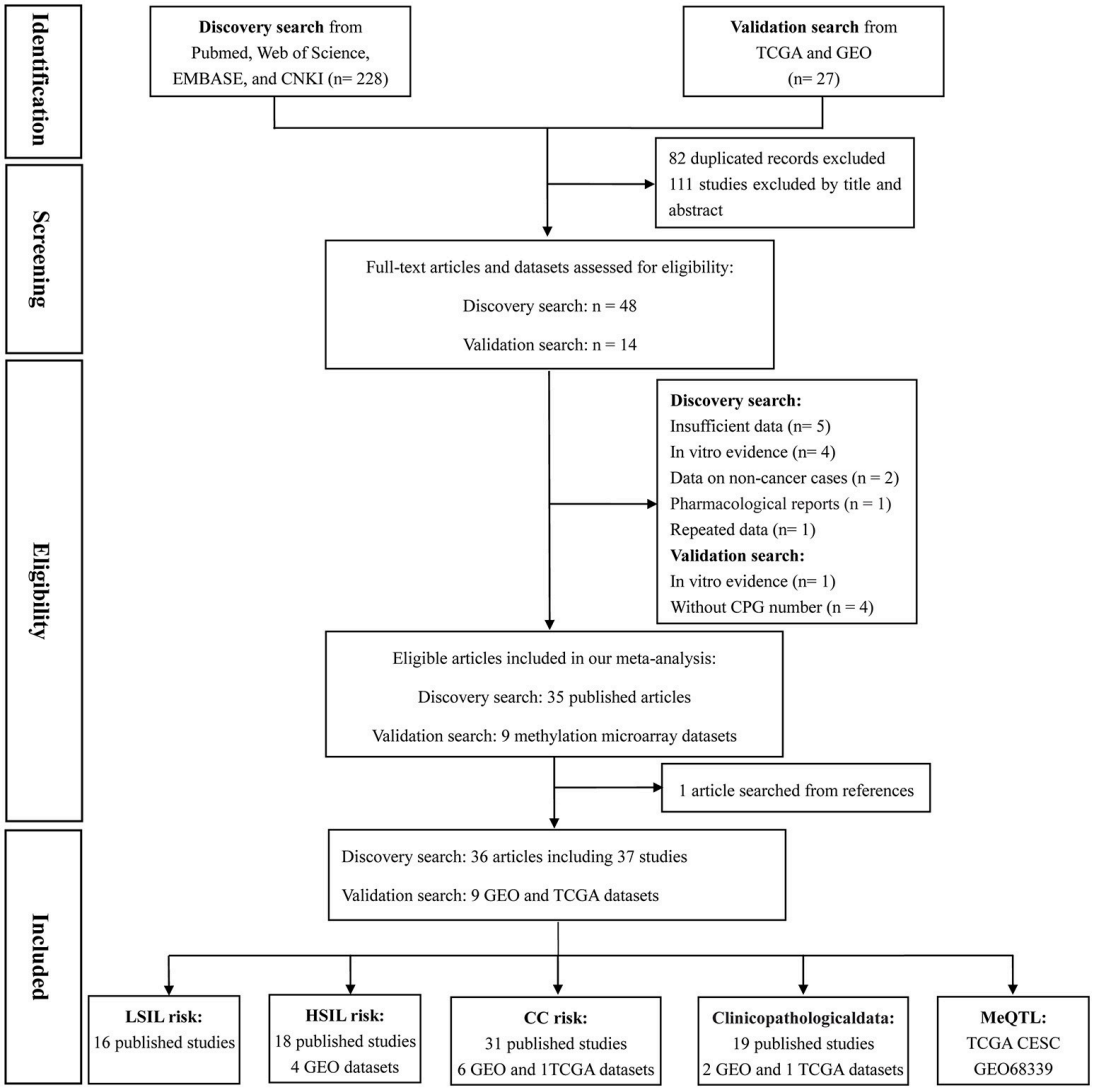
Flow chart of study selection process.

Based on the categorization of the 2001 Bethesda System (Solomon et al., 2002), the category of LSIL encompassed productive HPV infection, CIN1, and mild dysplasia; the diagnosis of HSIL corresponded to CIN2 and 3, moderate and extensive dysplasia, and CIS; CC included squamous cell carcinoma (SCC) and adenocarcinoma (AdC). According to these definitions and our literature search strategy, 48 published articles and 14 methylation datasets (from TCGA and GEO databases) were initially screened. Then, 18 of these studies were excluded due to incomplete (*n* = 5) or repeated data (*n* = 1), *in vitro* evidence (*n* = 5), pharmacological report (*n* = 1), research studying non-cancer specimens (*n* = 2), and datasets without CPG information (*n* = 4). In one remaining published article, methylation data from cervix and plasma were separately recorded (Yang et al., 2004). Manual search of references cited in literatures spotted one additional study (Widschwendter et al., 2004). Finally, a total of 45 reports, involving 37 published studies (Dong et al., 2001; Narayan et al., 2003; Gustafson et al., 2004; Reesink-Peters et al., 2004; Widschwendter et al., 2004; Yang et al., 2004, 2006, 2010; Feng et al., 2005, 2007; Kang et al., 2005, 2006; Jeong et al., 2006; Wisman et al., 2006; Henken et al., 2007; Shivapurkar et al., 2007; Kahn et al., 2008; Leung et al., 2008; Zhao et al., 2008; Flatley et al., 2009; Iliopoulos et al., 2009; Chaopatchayakul et al., 2010; Kim et al., 2010; Lim et al., 2010; Huang et al., 2011; Missaoui et al., 2011; Niyazi et al., 2012; Banzai et al., 2014; Kalantari et al., 2014; Li et al., 2015; Milutin Gasperov et al., 2015; Siegel et al., 2015; Sun et al., 2015; Yin et al., 2015; Jha et al., 2016; Bhat et al., 2017) and nine methylation datasets (Teschendorff et al., 2010, 2012; Guenin et al., 2012; Teschendorff and Widschwendter, 2012; Zhuang et al., 2012; Farkas et al., 2013; Lando et al., 2015), were included in the meta-analysis. The study selection process was shown in Figure 1. The study characteristics were listed in Table 1.

**Table 1:** Characteristics of included studies in this meta-analysis.

**No**.	**First author, Year**	**Country**	**Ethnicity**	**Study design**	**Sample size**				**Methylation detection method**	**Primer sets**	**Source of controls**	**Involved clincopathological features**	**Quality scores**
					**Control**	**CC**	**HSIL**	**LSIL**					
**PUBLISHED STUDIES SEARCHED FROM PUBMED, WEB OF SCIENCE, EMBASE, AND CHINA NATIONAL KNOWLEDGE INFRASTRUCTURE DATABASES**													
1	Dong et al., 2001	Korea	Asian	Case-control	24	53	–	–	MSP and sequencing	1	B	Histological type, grade, age	15
2	Narayan et al., 2003	Mix	Mix	Case-control	8	82	–	–	MSP	1	H	FIGO stage, histological type, therapeutic response, age, HPV	13
3	Gustafson et al., 2004	USA	Caucasian	Case-control	11	–	11	17	Nested MSP	1	H	–	11
4	Reesink-Peters et al., 2004	Netherlands	Caucasian	Case-control	41	48	–	–	qMSP	1	H	–	13
5	Widschwendter et al., 2004	Austria	Caucasian	Case-control	10	11	31	3	MSP	2	H		12
6	Yang et al., 2004	China	Asian	Case-control	100	85	–	–	MSP and sequencing	1	A	FIGO stage, histological type, grade	13
7	Yang et al., 2004^a^	China	Asian	Case-control	30	40	–	–	MSP and sequencing	1	H	–	13
8	Feng et al., 2005	Senegal	African	Case-control	142	92	46	39	MSP	1	M	–	10
9	Kang et al., 2005	Korea	Asian	Case-control	17	82	–	–	MSP	1	H	–	12
10	Jeong et al., 2006	Korea	Asian	Case-control	24	78	–	–	MSP	1	B	FIGO stage, histological type, tumor size, age	10
11	Kang et al., 2006	Korea	Asian	Case-only	–	92	–	–	MSP	1	–	Histological type	12
12	Wisman et al., 2006	Netherlands	Caucasian	Case-control	19	28	–	–	qMSP	1	H	Histological type	12
13	Yang et al., 2006	China	Asian	Case-only	–	127	–	–	MSP and sequencing	1	–	FIGO stage, histological type, grade	12
14	Feng et al., 2007^a^	Senegal	African	Case-control	16	63	25	9	MethyLight	3	B	HPV	10
15	Henken et al., 2007	Netherlands	Caucasian	Case-only	–	24	–	–	MS-MLPA	N	–	Histological type	13
16	Shivapurkar et al., 2007	USA	Caucasian	Case-control	12	45	23	–	qMSP	1	H	Histological type	12
17	Kahn et al., 2008	USA	Caucasian	Case-control	30	–	39	30	qMSP	4	H	–	12
18	Leung et al., 2008	China	Asian	Case-control	72	107	–	–	MSP	1	M	FIGO stage, histological type, therapeutic response, age, LNM	9
19	Zhao et al., 2008^b^	China	Asian	Case-control	20	52	–	–	MSP	1	B	FIGO stage, histological type, grade, LNM	9
20	Flatley et al., 2009	UK	Caucasian	Case-control	40	42	94	46	MSP	1	B	–	13
21	Iliopoulos et al., 2009	Greece, USA	Caucasian	Case-control	15	67	12	15	MethyLight	2	H	FIGO stage	12
22	Chaopatchayakul et al., 2010	Thailand	Asian	Case-control	28	85	–	–	MSP	1	H	FIGO stage, histological type, tumor size, therapeutic response, age	13
23	Kim et al., 2010	Korea	Asian	Case-control	41	69	67	32	Nested MSP	1	H	–	13
24	Lim et al., 2010	Singapore	Asian	Case-control	53	10	41	61	qMSP	1	H	–	13
25	Yang et al., 2010	Netherlands	Caucasian	Case-control	20	60	20	20	qMSP	1	B	FIGO stage, histological type, grade, tumor size, LNM, therapeutic response, HPV	10
26	Huang et al., 2011	China	Asian	Case-control	15	26	41	12	MSP	1	H	–	12
27	Missaoui et al., 2011	Tunisia	African	Case-control	8	14	16	12	MSP	2	H	–	12
28	Niyazi et al., 2012^b^	China	Asian	Case-control	30	30	30	30	MSP	1	B	–	9
29	Banzai et al., 2014	Japan	Asian	Case-control	24	53	22	–	MSP	N	H	Histological type	10
30	Kalantari et al., 2014	USA, Norway	Caucasian	Case-control	8	29	31	20	BSP	5	B	–	13
31	Li et al., 2015^b^	China	Asian	Case-control	90	100	–	–	MSP	1	B	FIGO stage, histological type, grade	10
32	Milutin Gasperov et al., 2015	Croatia	Caucasian	Case-control	40	10	81	40	MSP	1	H	–	10
33	Siegel et al., 2015	USA	Caucasian	Case-control	22	46	–	–	Pyrosequencing	N	H	FIGO stage	11
34	Sun et al., 2015	China	Asian	Case-control	48	45	103	54	HRM	6	B	–	10
35	Yin et al., 2015	China	Asian	Case-control	27	43	–	–	qMSP	7	B	–	10
36	Jha et al., 2016^a^	India	Asian	Case-only	–	27	–	–	MSP	1	–	FIGO stage	10
37	Bhat et al., 2017	India	Asian	Case-control	20	20	–	–	NGS	1	H	–	10
**QUANTITATIVE METHYLATION DATASETS FROM TCGA AND GEO DATABASES**													
38	TCGA CESC	USA	Mix	Case-control	3	307	–	–	450 K BeadChip	–	A	FIGO stage, histological type, grade, OS, DFS	14
39	GSE20080	UK	Caucasian	Case-control	30	–	18	–	27 K BeadChip	–	H	-	12
40	GSE30760	UK	Caucasian	Cohort	48	167	-	–	27 K BeadChip	–	M		12
41	GSE36637	Belgium	Caucasian	Case-control	4	5	–	–	27 K BeadChip	–	H	-	11
42	GSE37020	UK	Caucasian	Case-control	24	–	24	–	27 K BeadChip	–	B	-	12
43	GSE41384	Colombia	Mix	Case-control	3	3	10	3	27 K BeadChip	–	H		13
44	GSE46306	Sweden	Caucasian	Case-control	20	6	18	–	450 K BeadChip	–	H		13
45	GSE68339	Norway	Caucasian	Case-control	20	270	-	–	450 K BeadChip	–	H	FIGO stage	13
46	GSE99511	Netherlands	Caucasian	Case-control	28	4	36	–	450 K BeadChip	–	B	-	11

CC, cervical cancer; LSIL, low-grade squamous intra-epithelial lesion; HSIL, high-grade squamous intra-epithelial lesion; MSP, methylation-specific PCR; MS-MLPA, methylation specific-multiplex ligation-dependent probe amplification; BSP, bisulfite sequencing PCR; NGS, next generation sequencing; H, healthy controls; B, controls with benign cervical diseases; A, autologous controls; M, mixed controls.

a Three studies detected DAPK1 promoter hypermethylation in plasma, serum, and urine samples, the others used cervical tissues.

b Studies written in Chinese.

### Effect of *DAPK1* promoter hypermethylation on LSIL in meta-analyses of published studies

**Figure 2:**
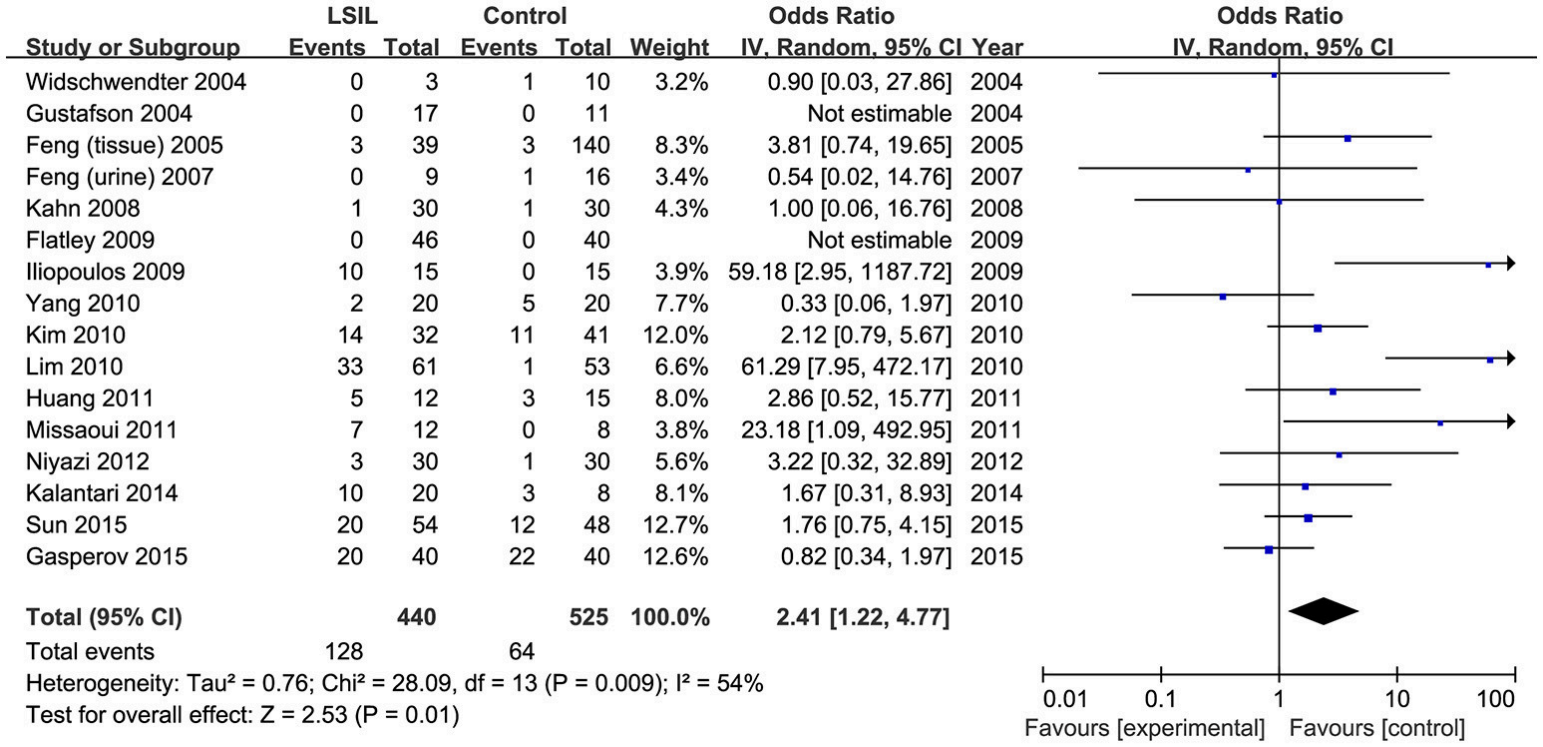
Funnel plots for the associations of *DAPK1* promoter hypermethylation with the risk of LSIL. The squares represent the ORs for individual studies. The size of the square reflects the weight of included studies. Bars represent the 95% confidence intervals (CIs). The center of the diamond represents the summary effect size. LSIL, low-grade intra-epithelial lesion.

A total of 440 LSIL patients and 525 controls, from 16 published studies, were combined to examine the effect of *DAPK1* promoter hypermethylation on LSIL (Figure 2). The pooled rate of *DAPK1* promoter hypermethylation was 27.5% (95%CI: 17.8–40.0%) in LSIL patients. *DAPK1* promoter hypermethylation conferred a 2.41-fold increased risk of LSIL (*P* = 0.012), with a moderate level of heterogeneity (*I*^2^ = 54%, Figure 2, Table 2). Galbraith plots identified two studies (Iliopoulos et al., 2009; Lim et al., 2010) as outliers and possible sources of heterogeneity (Figure S1A). After excluding these two studies, the association between *DAPK1* promoter hypermethylation and LSIL was still significant (OR = 1.55, *P* = 0.042), and the heterogeneity was effectively removed (*I*^2^ = 0%). In subgroup analyses, *DAPK1* promoter hypermethylation was also associated with LSIL risk in Asians, high-quality reports, and studies using healthy controls (Table 2).

**Table 2:** Pooled results for the association of *DAPK1* promoter hypermethylation with LSIL risk.

**Comparisons**	**Studies (*N*)**	**Sample size (CC/controls)**	**Heterogeneity**		***P*_meta-regression_**	**Model^a^**	**Effect size**	
			**I^2^(%)**	***P*_Q-test_**			**OR (95% CI)**	***P***
Total	16	440/525	54	0.009	–	R	2.41 (1.22–4.77)	0.012
Ethnicity					0.702			
Asian	5	189/187	61	0.037		R	3.65 (1.33–10.01)	0.012
Caucasian	8	191/174	45	0.104		F	1.01 (0.52–1.95)	0.981
Other ethnicities	3	60/164	25	0.263		F	3.89 (0.74–20.50)	0.109
Source of controls					0.380			
Healthy	10	231/239	66	0.003		R	3.53 (1.17–10.62)	0.025
Non-healthy^b^	6	209/286	10	0.349		F	1.67 (0.90–3.10)	0.109
Study quality					0.093			
High (>11)	9	231/220	55	0.029		R	4.83 (1.61–14.44)	0.005
Low (≤ 11)	7	209/305	21	0.279		F	1.26 (0.74–2.12)	0.396
Primer set					0.743			
1	10	351/438	64	0.007		R	2.22 (0.99–4.94)	0.052
2–7	6	89/87	38	0.153		F	2.82 (0.97–8.20)	0.058

N, number; F, fixed-effects model; R, Random-effects model; LSIL, low-grade squamous intra-epithelial lesion.

a If significant heterogeneity was found (I^2^ ≥ 50% or P_Q−test_ ≤ 0.1), a random-effects model with the inverse variance method was applied.

b Non-healthy controls included autologous controls, controls with benign gynecological diseases, and mixed controls.

### Effect of *DAPK1* promoter hypermethylation on HSIL in meta-analyses of published studies

**Figure 3:**
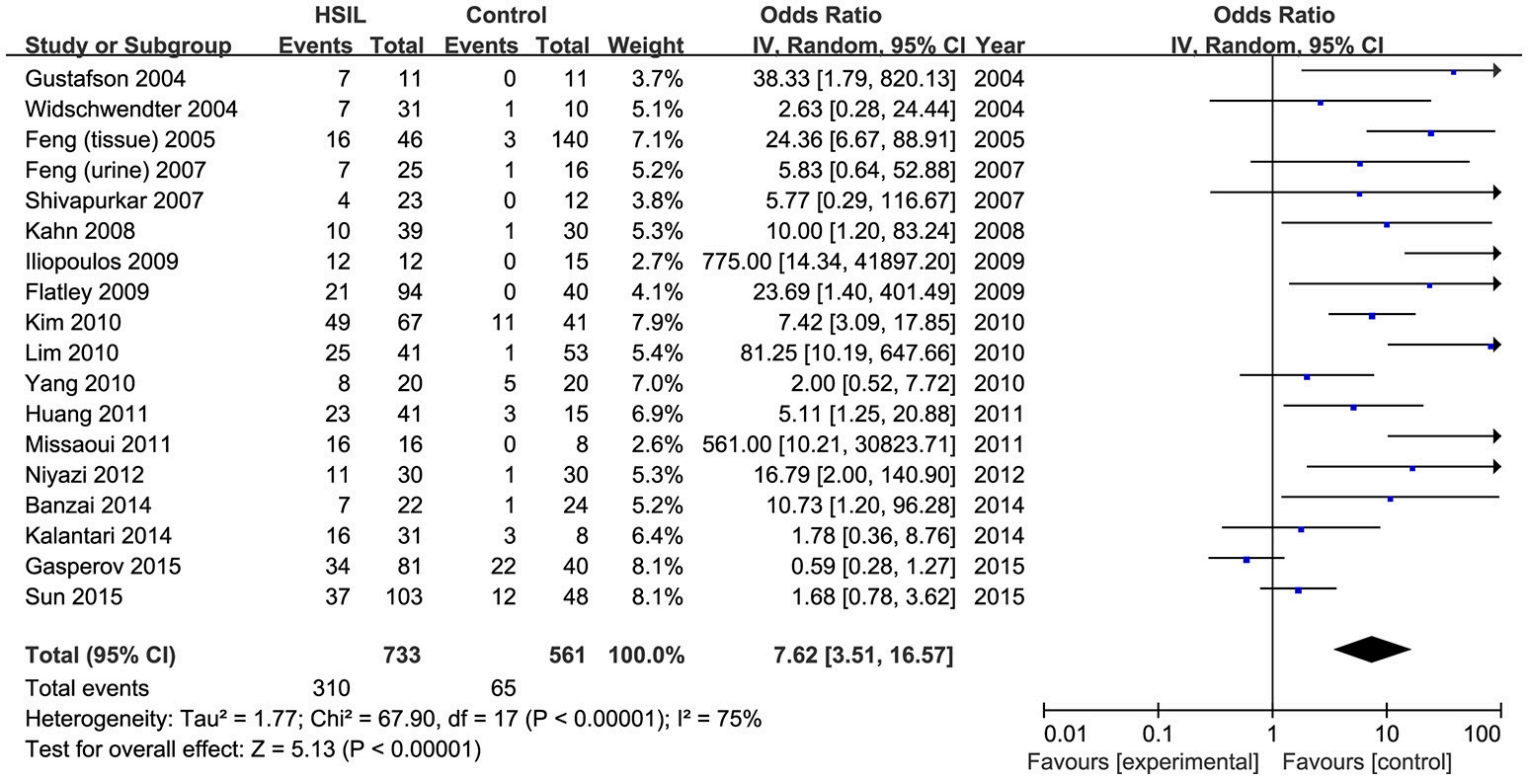
Funnel plots for the associations of *DAPK1* promoter hypermethylation with the risk of HSIL. HSIL, high-grade intra-epithelial lesion.

Eighteen published studies, with 733 HSIL patients and 561 controls, were included for analyzing the effect of *DAPK1* promoter hypermethylation on HSIL (Figure 3). *DAPK1* promoter was found to be hypermethylated in 42.2% (95% CI: 33.4–51.5%) of HSIL patients. There was a significant association between *DAPK1* promoter hypermethylation and increased HSIL risk in the overall comparison (OR = 7.62, *P* < 0.001) and in all subgroups (Figure 3, Table 3). To identify the origin of high heterogeneity in the overall comparison (*I*^2^ = 75%), we performed a meta-regression procedure, which identified study quality as a significant source of heterogeneity (*P* = 0.004), accounting for 68.7% of total variance. Through analysis of subgroup heterogeneity, we found that overall heterogeneity was substantially reduced in subgroups of either high-quality studies (*I*^2^ = 5%) or low-quality reports (*I*^2^ = 11%), further supporting the results of meta-regression (Table 3).

**Table 3:** Pooled results for the association of *DAPK1* promoter hypermethylation with HSIL risk.

**Comparisons**	**Studies (*N*)**	**Sample size (HSIL/controls)**	**Heterogeneity**		***P*_meta-regression_**	**Model^a^**	**Effect size**	
			***I*^2^(%)**	***P*_Q-test_**			**OR (95% CI)**	***P***
Total	18	733/561	75	<0.001	–	R	7.62 (3.51–16.57)	<0.001
Ethnicity					0.610			
Asian	6	304/211	72	0.003		R	7.76 (2.76–21.86)	<0.001
Caucasian	9	342/186	71	0.001		R	4.95 (1.50–16.31)	0.008
Other ethnicities	3	87/164	49	0.140		F	21.73 (7.41–63.70)	<0.001
Source of controls					0.487			
Healthy	11	409/275	78	<0.001		R	10.53 (3.40–32.59)	<0.001
Non-healthy^b^	7	324/286	71	0.004		R	5.10 (1.67–15.54)	0.004
Study quality					**0.004**			
High (>11)	11	420/248	5	0.761		F	8.09 (4.71–13.88)	<0.001
Low (≤ 11)	7	313/313	11	0.312		F	2.10 (1.35–3.27)	<0.001
Primer set					0.90			
1	10	454/402	81	<0.001		R	8.14 (2.68–24.69)	<0.001
2-7	7	257/135	67	0.006		R	6.99 (1.87–26.08)	0.004

N, number; F, fixed-effects model; R, Random-effects model; HSIL, high-grade squamous intra-epithelial lesion.

a If significant heterogeneity was found (I^2^ ≥ 50% or P_Q−test_ ≤ 0.1), a random-effects model with the inverse variance method was applied.

b Non-healthy controls included autologous controls, controls with benign gynecological diseases, and mixed controls.

Bold values indicate significant results with P < 0.05 in meta-regression.

### Effect of *DAPK1* promoter hypermethylation on CC in meta-analyses of published studies

**Figure 4:**
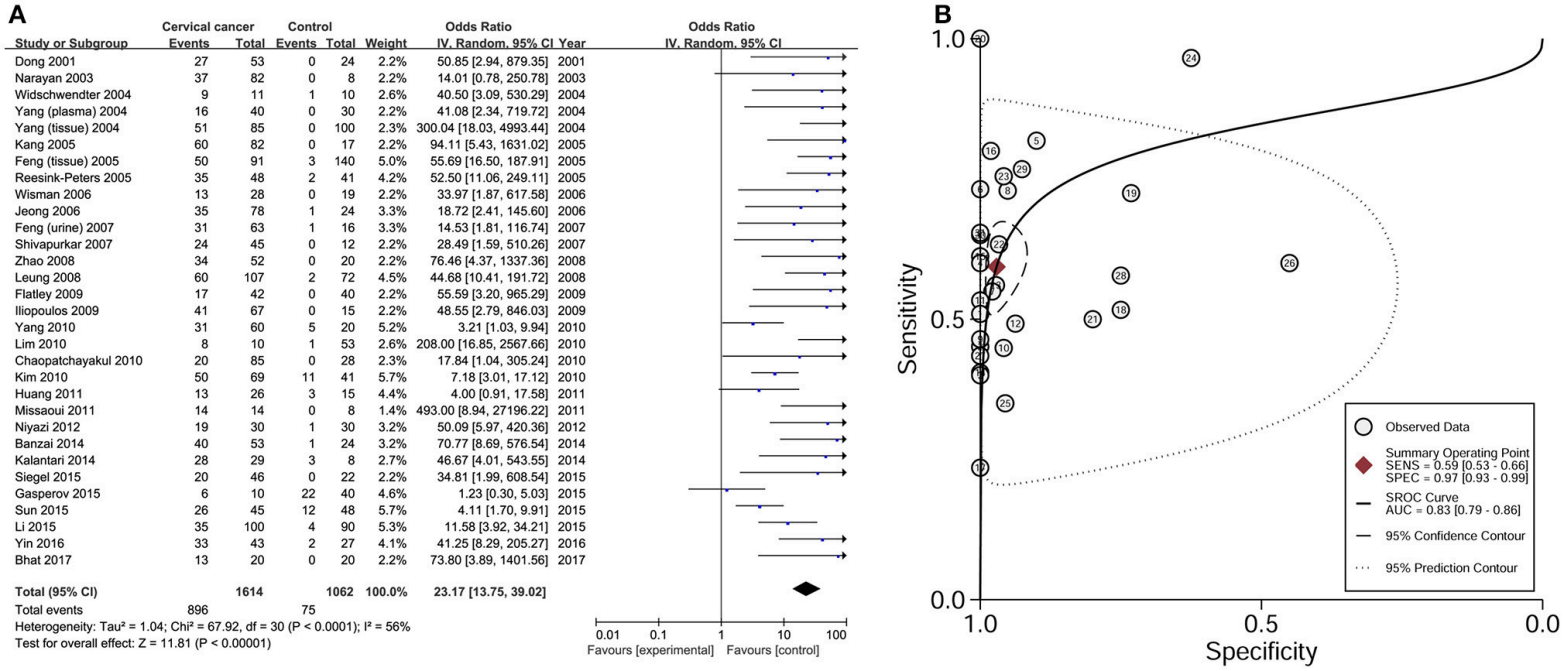
Risk assessment and diagnostic value of *DAPK1* promoter hypermethylation in CC. **(A)** Meta-analyses for the association between *DAPK1* promoter hypermethylation and CC risk; **(B)** SROC curves showing the diagnostic value of *DAPK1* methylation detection in CC. CC, cervical cancer; SROC, Summary receiver operating characteristic.

Data from 31 studies with 1614 CC patients and 1062 controls were combined to appraise the association between *DAPK1* methylation status and CC (Figure 4A). In CC patients, the pooled rate of *DAPK1* promoter hypermethylation reached 57.0% (51.3–62.5%). *DAPK1* promoter hypermethylation was constantly associated with an increased risk of CC in the overall comparison (OR = 23.17, *P* < 0.001, Figure 4A) as well as in subgroup analyses (Table 4). Since moderate heterogeneity was observed in the overall comparison (*I*^2^ = 56%), a Galbraith plot was depicted, spotting three outliers (Yang et al., 2010; Milutin Gasperov et al., 2015; Sun et al., 2015) as major sources of heterogeneity (Figure S1B). Exclusion of these three studies led to a decrease in *I*^2^ value from 56 to 16%, accompanied by a significant association between *DAPK1* promoter hypermethylation and increased CC risk (OR = 25.38, *P* < 0.001). Meta-regression suggested that study quality explained 31.6% of total heterogeneity, with a *P* = 0.049 (Table 4).

**Table 4:** Pooled results for the association of *DAPK1* promoter hypermethylation with CC risk.

**Comparisons**	**Studies (*N*)**	**Sample size (CC/controls)**	**Heterogeneity**		***P*_meta-regression_**	**Model^a^**	**Effect size**	
			***I*^2^(%)**	***P*_Q-test_**			**OR (95% CI)**	***P***
Total	31	1614/1062	56	<0.001	–	R	23.17 (13.75–39.02)	<0.001
Ethnicity					0.978			
Asian	17	978/663	54	0.004		R	24.47 (12.72–47.06)	<0.001
Caucasian	10	386/227	62	0.005		R	17.79 (5.93–53.34)	<0.001
Other ethnicities	4	250/172	6	0.361		F	40.77 (15.63–106.29)	<0.001
Source of controls					0.931			
Healthy	17	718/392	52	0.006		R	23.43 (10.61–51.77)	<0.001
Non-healthy^b^	14	896/670	62	0.001		R	23.77 (11.55–48.96)	<0.001
Study quality					**0.049**			
High (> 11)	17	836/476	16	0.269		F	33.27 (19.81-55.88)	<0.001
Low (≤ 11)	14	778/586	69	<0.001		R	14.88 (7.05-31.42)	<0.001
Primer set					0.908			
1	22	1243/884	59	<0.001		R	22.04 (11.76-41.33)	<0.001
2-6	7	272/132	58	0.027		R	24.63 (7.46-81.27)	<0.001

N, number; F, fixed-effects model; R, Random-effects model; CC, cervical cancer.

a When significant heterogeneity was found (I^2^ ≥ 50% or P_Q−test_ ≤ 0.1), a random-effects model with the inverse variance method was used to pool the results; otherwise, a fixed-effects model was applied.

b Non-healthy controls included autologous controls, controls with benign gynecological diseases, and mixed controls.

Bold values indicate significant results with P < 0.05 in meta-regression.

To assess the diagnostic value of *DAPK1* methylation status in CC, we constructed a SROC curve using the random-effects model, which showed a high specificity of 97% and a moderate sensitivity of 59%. Moreover, the AUC reached 83% (Figure 4B), supporting a potential ability of *DAPK1* methylation detection to discriminate CC from controls.

### Correlations of *DAPK1* promoter hypermethylation with clinicopathological features of CC

By combining the methylation data from 19 studies with 1315 CC patients, we analyzed the effect of DAPK1 promoter hypermethylation on clinicopathological features of CC (Dong et al., 2001; Narayan et al., 2003; Yang et al., 2004, 2006, 2010; Jeong et al., 2006; Kang et al., 2006; Wisman et al., 2006; Feng et al., 2007; Henken et al., 2007; Shivapurkar et al., 2007; Leung et al., 2008; Zhao et al., 2008; Iliopoulos et al., 2009; Chaopatchayakul et al., 2010; Kalantari et al., 2014; Li et al., 2015; Siegel et al., 2015; Jha et al., 2016). As presented in Table 5, patients with SCC had higher frequencies of *DAPK1* promoter hypermethylation than those with AdC (OR = 3.53, *P* < 0.001, Figure S2); *DAPK1* promoter hypermethylation was significantly correlated with advanced International Federation of Gynecology and Obstetrics (FIGO) stage of CC (OR = 2.15, *P* = 0.003, Figure S3), but not with histological grade, lymph node metastasis, HPV infection, age at diagnosis, and therapeutic responses.

**Table 5:** Pooled results for the associations between *DAPK1* promoter hypermethylation and clincopathological features of CC.

**Clincopathological features**	**Studies (*N*)**	**Patients (*N*)**	**Heterogeneity**		**Model^a^**	**Effect size**	
			***I*^2^(%)**	***P*_Q–test_**		**OR (95% CI)**	***P***
Histological type (SCC vs. AdC)	15	1071	0	0.839	F	**3.53 (2.55–4.90)**	<**0.001**
FIGO stage (III + IV vs. I + II)	12	906	52	0.017	R	**2.15 (1.31–3.56)**	**0.003**
Histological grade (G3 vs. G1 + G2)	4	264	0	0.766	F	1.12 (0.66-1.88)	0.681
Tumor size (≥ 4 cm vs. < 4 cm)	3	222	0	0.896	F	1.15 (0.64-2.08)	0.638
Lymph node metastasis (Yes vs. No)	3	120	5	0.347	F	1.31 (0.53-3.23)	0.552
HPV infection (Positive vs. Negative)	4	323	0	0.872	F	1.51 (0.85-2.66)	0.158
Therapeutic response (Yes vs. No)^b^	4	259	82	<0.001	R	0.71 (0.18-2.80)	0.629
Age at diagnosis (> 50 vs. ≤ 50)	5	405	0	0.947	F	1.26 (0.84-1.90)	0.270

N, number; SCC, squamous cell carcinoma; AdC, adenocarcinoma; F, fixed-effects model; R, random-effects model.

a When significant heterogeneity was found (I^2^ ≥ 50% or P_Q−test_ ≤ 0.1), a random-effects model with the inverse variance method was used to pool the results; otherwise, a fixed-effects model was applied.

b Therapeutic response included responses to radiotherapy, concurrent chemoradiotherapy, radical hysterectomy with pelvic lymph node dissection, or transabdominal hysterectomy.

Bold values indicate significant results with P < 0.05.

### Validation by quantitative methylation data from TCGA and GEO databases

**Figure 5:**
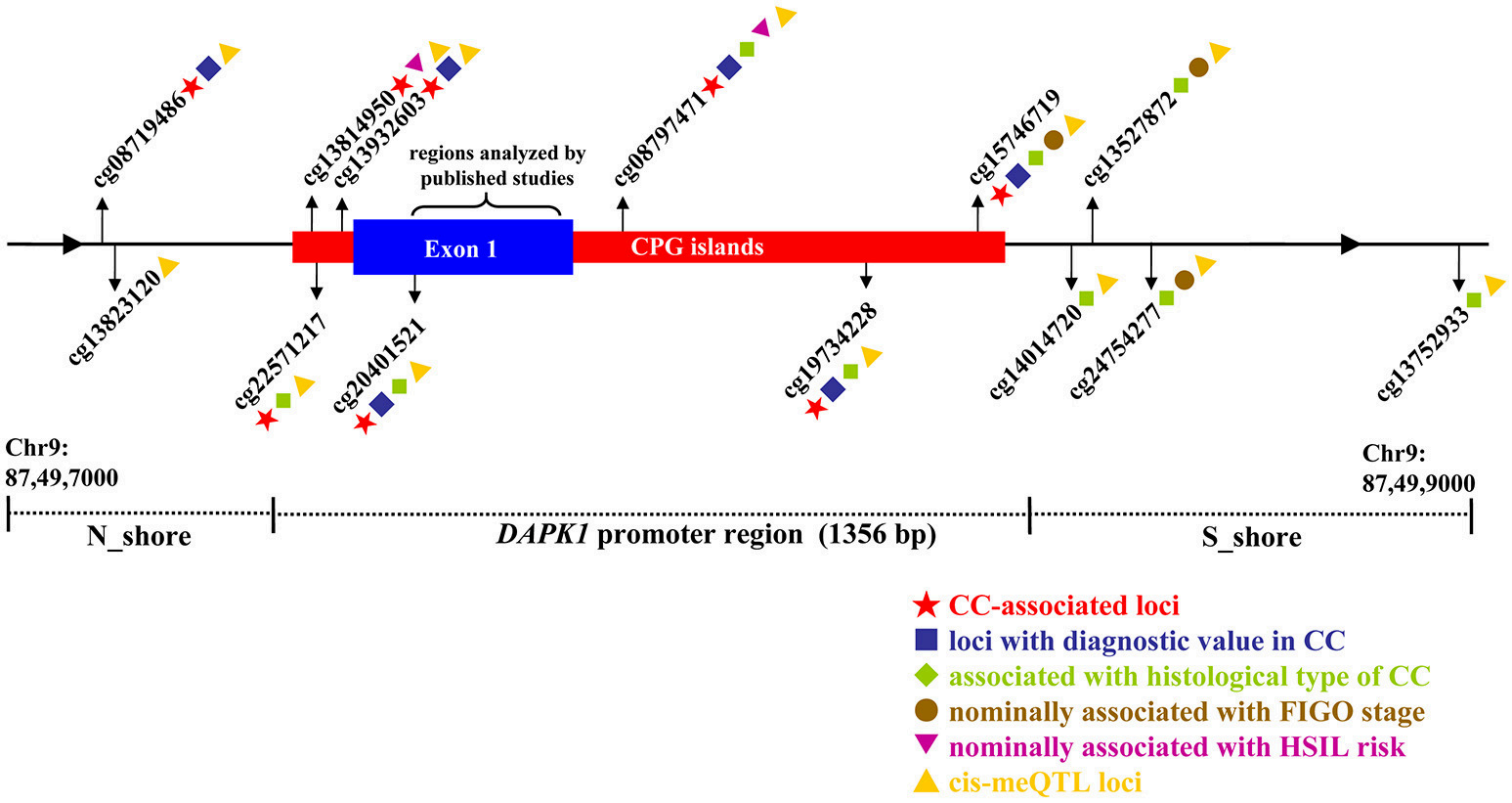
Illustrative map for the associations of 13 CPG sites in *DAPK1* promoter with CC risk, histological type of CC, FIGO stage of CC, HSIL risk, and *DAPK1* mRNA expression in a pooled analysis of nine TCGA and GEO datasets.

To validate the significant results of published studies, seven TCGA and GEO datasets (TCGA CESC, GSE30760, GSE36637, GSE41384, GSE46306, GSE68339, and GSE99511), involving 643 CC patients and 245 controls, were pooled to analyze the associations of 13 CPG sites in *DAPK1* with CC. Using a genome-wide significance threshold of *P* < 10^−7^, 8 of 13 CPG sites in *DAPK1* were identified as differentially methylated between CC patients and controls (Table 6, Figure 5). Out of eight CC-associated sites, six had pooled sensitivities of 70–81%, pooled specificities of 74–90%, and AUCs of 0.74–0.95 in SROC curves (Table 6), validating the diagnostic value of *DAPK1* methylation status in CC.

**Table 6:** Risk assessment and diagnostic value of 13 CPG sites of *DAPK1* promoter for CC.

**CPG sites**	**Location^a^**	**CPG features^b^**	**Studies *N*^c^**	**Sample size (CC/controls)**	**Risk assessment of CPG sites for CC**					**Diagnostic value of CPG sites in CC**			
					**Heterogeneity**		**Effect size**			**Cut-off β value**	**Specificity (%)**	**Sensitivity (%)**	**AUC**
					***I*^2^(%)**	***P*_Q–test_**	**Model^d^**	**SMD (95%CI)**	***P***				
cg08719486	chr9: 87497186	N_shore	7	643/245	80	<0.001	R	**2.44 (2.16, 2.72)**	**1.08** × **10**^−104^	**0.566**	**90**	**81**	**0.95**
cg13823120	chr9: 87497210	N_shore	4	587/71	55	0.083	R	1.08 (0.43, 1.74)	0.001	0.429	85	60	0.77
cg13814950	chr9: 87497600	Island	7	643/245	9	0.357	F	**0.86 (0.61, 1.10)**	**1.07** × **10**^−20^	0.079	97	33	0.63
cg22571217	chr9: 87497604	Island	7	643/245	42	0.108	F	**0.87 (0.63, 1.12)**	**7.37**× **10**^−20^	0.097	97	34	0.70
cg13932603	chr9: 87497600	Island	4	587/71	0	0.892	F	**0.85 (0.49, 1.22)**	**3.40** × **10**^−11^	**0.062**	**74**	**71**	**0.74**
cg20401521	chr9: 87497796	Island	4	587/71	0	0.769	F	**1.17 (0.80, 1.54)**	**5.66** × **10**^−13^	**0.070**	**76**	**70**	**0.76**
cg08797471	chr9: 87498205	Island	7	643/245	51	0.059	R	**1.01 (0.57, 1.45)**	**1.19** × **10**^−45^	**0.162**	**75**	**78**	**0.80**
cg19734228	chr9: 87498678	Island	7	643/245	38	0.142	F	**1.13 (0.89, 1.38)**	**1.59** × **10**^−50^	**0.173**	**84**	**73**	**0.87**
cg15746719	chr9: 87498898	Island	7	643/245	77	<0.001	R	**1.67 (0.95, 2.40)**	**3.05** × **10**^−58^	**0.166**	**80**	**72**	**0.83**
cg14014720	chr9: 87499083	S_shore	4	587/71	76	0.006	R	0.59 (−0.29, 1.47)	0.190	0.200	58	51	0.47
cg13527872	chr9: 87499122	S_shore	2	309/31	0	0.581	F	−0.49 (−1.27, 0.28)	0.212	0.403	56	51	0.46
cg24754277	chr9: 87499241	S_shore	7	643/245	79	<0.001	R	0.76 (0.08, 1.44)	0.028	0.344	61	62	0.74
cg13752933	chr9: 87499840	S_shore	4	587/71	76	0.006	R	0.13 (−0.75, 1.00)	0.776	0.334	61	43	0.31

*N, number; CC, cervical cancer; SMD, standardized mean differences; AUC, area under the curve*.

a Information for chromosome position is based on NCBI genome build 38.2.°

b According to the TCGA data user's guide, Island means the start coordinates of the CPG island; N_shore means 0-2 kb upstream from the position of the CPG island; S_shore means 0-2 kb downstream from the position of the CPG island.

c TCGA CESC, GSE68339, GSE99511, and GSE46306 used the Illumina 450 K BeadChip, which included methylation probes of all 13 CPG sites in DAPK1 promoter; another three datasets (GSE30760, GSE36637, and GSE41384) used 27 K BeadChip, which detected seven CPG sites, including cg08719486, cg13814950, cg22571217, cg08797471, cg19734228, cg15746719, and cg24754277.

d When significant heterogeneity was found (I^2^ ≥ 50% or P_Q−test_ ≤ 0.1), a random-effects model with the inverse variance method was used to pool the results; otherwise, a fixed-effects model was applied.

Bold values indicate significant results with P values < 10-7.

**Figure 6:**
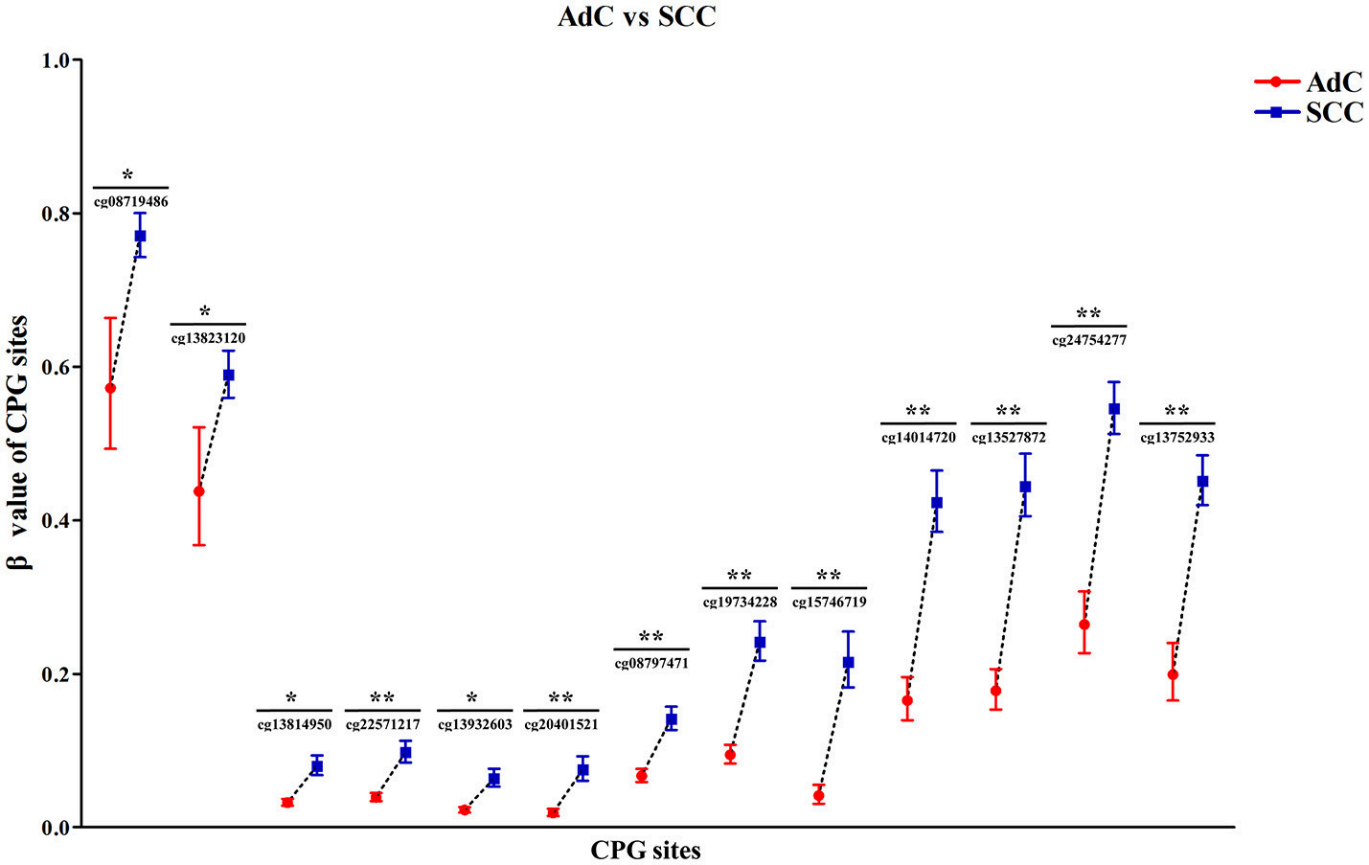
Significant differences in 13 CPG sites of *DAPK1* between SCC (*n* = 254) and AdC (*n* = 53) in the TCGA CESC dataset. Methylation values of 13 CPG sites were expressed as genometric mean (95%CI) due to ln-transformation before analysis. *P*-values were obtained from the Mann–Whitney U test. **P < 10^−7^; *P < 0.01.

Then, by searching the TCGA CESC dataset, we achieved the histological data of 307 CC tissues, and found that methylation levels at all 13 CPG sites were constantly higher in SCC than in AdC (*P* < 0.05). Out of 13 associated loci, 9 showed genome-wide significance results with *P-*values ranging from 1.72 × 10^−8^ to 1.12 × 10^−15^ (Figure 6, Table S3), supporting the effect of *DAPK1* promoter hypermethylation on histological type of CC. However, no CPG sites in *DAPK1* were correlated with histological grade, DFS and OS of CC patients (Tables S4, S5).

Three datasets, including TCGA CESC, GSE30760, and GSE68339, recorded the FIGO stage of 560 CC patients. Meta-analyses of these raw data suggested that methylation of four CPG sites in *DAPK1* had nominally positive effects on advanced FIGO stage, with *P-*values (ranging from 0.047 to 8.53 × 10^−5^) that did not exceed the genome-wide significance threshold (Table S6).

Finally, in meta-analysis of five datasets (GSE20080, GSE37020, GSE41384, GSE46306, and GSE99511) involving 106 HSIL patients and 105 controls, we identified two CPG sites nominally associated with HSIL risk, but the *P-*values did not reach the genome-wide significance level (cg13814950: *P* = 0.030; cg08797471: *P* = 0.003, Table S7).

### MeQTL analyses for 13 CPG sites in *DAPK1*

To verify the silenced impact of *DAPK1* promoter methylation on gene expression, meQTL analyses for *DAPK1* were performed by integrating the methylation and RNA-Seq data from GSE68339 and TCGA CESC datasets. In GSE68339 with 121 CC patients, all 13 CPG sites in *DAPK1* contributed to down-regulation of *DAPK1* mRNA expression, with *r*-coefficients ranging from −0.211 to −0.507 (*P*-values: 0.020–2.84 × 10^−9^, Table S8). Then, the TCGA CESC dataset, with a larger sample size of 309 cervical tissues, was used for replicating the above results. As expected, all 13 CPG sites were considered as cis-meQTL loci, with more significant impacts on silencing *DAPK1* mRNA expression (*r*-coefficients: −0.233 ~ − 0.547; *P-*values: 3.51 × 10^−5^ − 1.55 × 10^−25^, Table S8).

### Sensitivity and publication bias of meta-analyses

In sensitivity analyses, sequential removal of individual studies had no significant impact on the summary effect size in all comparisons (Figure S4), suggesting the stability of meta-analyses. Relatively symmetrical funnel plots (Figure S5), combined with non-significant results of the Egger 's test, indicated a lack of publication bias in all comparisons.

## Discussion

The silencing of *DAPK1* by promoter hypermethylation has been long linked to CC, but the established data showed a varied range of *DAPK1* promoter hypermethylation rates (24–100%) in cancer tissues (Chaopatchayakul et al., 2010; Missaoui et al., 2011) and inconsistent association results. Thus, Wentzensen et al. first conducted a systematical review of 18 heterogenous studies, which identified *DAPK1* as the second most frequently methylated gene in CC (Wentzensen et al., 2009). Then, two meta-analyses in 2014–2015, which included 15 and 20 case-control studies, respectively, consistently suggested a significant association between *DAPK1* promoter hypermethylation and CC (Xiong et al., 2014; Agodi et al., 2015). However, the following issues were still not fully summarized, promoting us to perform this updated meta-analysis. First, there were more studies investigating *DAPK1* promoter hypermethylation with CC risk in different populations since 2015. Second, most established reviews only focused on the epigenetic impact of *DAPK1* on CC risk, but the associations of *DAPK1* promoter hypermethylation with clinicopathological features and diagnostic value of CC were not summarized. Finally, *DAPK1* methylation status in the progression of SIL to CC should be analyzed, given the consecutive passages in cervical oncogenesis.

Therefore, by conducting the updated meta-analysis of 37 published studies, we first showed that the rate of *DAPK1* promoter hypermethylation increased with lesion severity, from 27.5% in LSIL tissues, 42.2% in HSIL tissues to 57.0% in CC specimens, and that *DAPK1* promoter hypermethylation progressively increased the risk of LSIL by 2.41-fold, HSIL by 7.62-fold, and CC by 23.17-fold. Then, SROC curves suggested a potential diagnostic value of *DAPK1* promoter hypermethylation in CC, with a large AUC of 83%, a high specificity of 97%, and a moderate sensitivity of 59%. Finally, *DAPK1* promoter hypermethylation was found to be associated with two clinicopathological features, i.e., histological type and FIGO stage of CC. These results were consistent with previous *in vitro* evidence that *DAPK1* methylation rates were gradually increased in consecutive stages from immortalization, anchorage independence, to tumorigenicity during carcinogenesis of HPV-transfected cells (Henken et al., 2007), suggesting the vital roles of *DAPK1* promoter hypermethylation in cancer progression. Notably, in both Asians and Caucasians, we observed a similar increasing trend of *DAPK1* promoter hypermethylation rates from LSIL, HSIL, to CC (Figure S6), further pinpointing the general effects of *DAPK1* promoter hypermethylation on lesion severity across ethnicities.

In meta-analyses of published studies, moderate-to-high levels of heterogeneity were observed for comparisons of *DAPK1* promoter hypermethylation with LSIL, HSIL, and CC. Thus, the methylation data were first combined using a random-effects model, which weighted a conservative summary effect estimate after adjusting for the inter-study variances. Then, the possible sources of heterogeneity were analyzed by three statistical approaches, including meta-regression and subgroup analyses to identify the confounding factors associated with observed heterogeneity, and Galbraith plots to visualize the contributions of individual studies to overall heterogeneity. In the comparison between *DAPK1* promoter hypermethylation and HSIL, meta-regression and subgroup analyses consistently suggested that study quality, assessed by our quality scoring scale, was the major origin of moderate heterogeneity; while Galbraith plots spotted two studies contributing to moderate heterogeneity for *DAPK1* promoter hypermethylation and LSIL (Iliopoulos et al., 2009; Lim et al., 2010), and at least three studies involving high heterogeneity for *DAPK1* promoter hypermethylation and CC (Yang et al., 2010; Milutin Gasperov et al., 2015; Sun et al., 2015). Notably, these five studies were all scored as low-quality reports, with some common flaws including lack of biospecimen information (Yang et al., 2010; Milutin Gasperov et al., 2015; Sun et al., 2015), lack of records on clincopathological data (Lim et al., 2010; Milutin Gasperov et al., 2015; Sun et al., 2015), and different primer sets used for methylation detection (Iliopoulos et al., 2009; Sun et al., 2015). Moreover, three methylation detection methods, including MSP, quantitative MSP, and high resolution melting analyses were applied in the five outliers, inducing potential heterogeneity resulting from inconsistent methylation-detected signals across different methodologies. Therefore, subsequent studies, with more scientific reporting fashions for sample materials, clinical data, and methylation detection, may help to strengthen the consistency of study results for *DAPK1* promoter hypermethylation and cervical neoplasia.

By reviewing the study characteristics, we found that *DAPK1* methylation detection in published literatures was mainly based on methylation specific PCR (MSP), which was a qualitative method relying on two primer sets to discriminate between methylated and unmethylated alleles (Umer and Herceg, 2013). However, at least seven primer designs, which analyzed different CPG regions in *DAPK1* (Table S1), were observed in included studies, causing the difficulty in interpreting the pooled results and potential publication bias. Moreover, the epigenetic silencing of *DAPK1* was primarily reported *in vitro* (SiHa and HeLa cell lines) (Narayan et al., 2003; Leung et al., 2008), but barely replicated in CC tissues. So, to better validate the epigenetic effect of *DAPK1*, 13 CPG sites, covering all the CPG islands investigated by literatures, were analyzed by extracting the methylation microarray datasets from TCGA and GEO databases. Consistent with the pooled results of published studies, we identified eight CC-associated CPG sites and nine loci with higher methylation levels in SCC than in AdC. Furthermore, in contrast to a moderate sensitivity (59%) calculated from qualitative data of published studies, SROC curves of quantitative methylation datasets screened six CPG sites with stronger sensitivities of up to 81% and acceptable specificities of 74–90%, suggesting a better ability of quantitative *DAPK1* methyaltion detection to predict CC. Finally, meQTL analyses of two independent cohorts constantly suggested that all 13 CPG sites contributed to down-regulation of *DAPK1* mRNA expression in CC tissues. All these results together provide reliable evidence that the epigenetic silencing of *DAPK1* is a predictive marker of CC, especially of SCC. However, only four CPG sites in *DAPK1* exhibited nominal associations with advanced FIGO stage of CC, suggesting the exaggerated observation for *DAPK1* promoter hypermethylation and FIGO stage in published studies and the necessity of validation by other data sources.

The meta-analyses had some limitations. First, based on a small sample size of 106 HSIL patients and 105 controls, meta-analyses of four quantitative methylation datasets only screened two CPG sites nominally associated with HSIL. This finding was not consistent with the pooled results of 18 published studies (733 HSIL patients and 561 controls), which showed a substantially (OR = 7.62) increased risk of HSIL for *DAPK1* promoter hypermethylation. Larger studies with quantitative methylation data are needed to resolve this controversy. Second, most included studies used retrospective designs (case-control, case-only studies), some of which were hospital-based, so selection bias may be inevitable in the meta-analyses.

In summary, the present study is the first meta-analysis that integrates the data from published studies and publicly available datasets to assess the exact roles of *DAPK1* promoter hypermethylation in cervical neoplasia. We suggests that *DAPK1* promoter hypermethylation down-regulates *DAPK1* mRNA expression, and progressively increases the risk of LSIL, HSIL, and CC. *DAPK1* methylation detection exhibits a promising diagnostic value for CC, especially for SCC.

## Author contributions

XW and LM conceived and designed the experiments. NC and XL conducted the literature search, study selection, and data extraction. SG and QZ performed the quality assessment for included studies. JM and JZ conducted statistical analyses. XW wrote the manuscript.

### Conflict of interest statement

The authors declare that the research was conducted in the absence of any commercial or financial relationships that could be construed as a potential conflict of interest.
