# Supplementary material for: Identification of DAPK1 Promoter Hypermethylation as a Biomarker for Intra-Epithelial Lesion and Cervical Cancer: A Meta-Analysis of Published Studies, TCGA, and GEO Datasets
Source: Front Genet. 2018 Jul 17;9:258. doi: 10.3389/fgene.2018.00258 (PMC6056635; doi:10.3389/fgene.2018.00258)
Supplement: Supplementary file 1 [file Data_Sheet_1.DOCX]

**Supplementary Materials**

**Identification of *DAPK1* Promoter Hypermethylation as a Biomarker for Intra-Epithelial Lesion and Cervical Cancer: A Meta-Analysis of Published Studies, TCGA, and GEO Datasets**

Table S1..........................................................................................................................2

Table S2..........................................................................................................................3

Table S3..........................................................................................................................4

Table S4..........................................................................................................................4

Table S5..........................................................................................................................5

Table S6..........................................................................................................................6

Table S7..........................................................................................................................7

Table S8..........................................................................................................................7

Figure S1........................................................................................................................8

Figure S2........................................................................................................................9

Figure S3........................................................................................................................9

Figure S4......................................................................................................................10

Figure S5......................................................................................................................11

Figure S6......................................................................................................................12

| **Table S1:** Seven pairs of primer sets used in 37 published studies. | | | | | | |
| --- | --- | --- | --- | --- | --- | --- |
| Primer set | Primer sequence | | Length (bp) | Detected region | Method | Ref No. |
| 1 | M | Forward: GGATAGTCGGATCGAGTTAACGTC | 98 | Chr9: 87,497,882-87,497,987 | MSP, qMSP, NGS, | [7, 26, 28-36, 38, 39, 41-44, 46-50, 52, 53, 55-57, 60, 61] |
|  |  | Reverse: CCCTCCCAAACGCCGA |  |  |  |  |
|  | U | Forward GGAGGATAGTTGGATTGAGTTAATGTT | 106 |  |  |  |
|  |  | Reverse: CAAATCCCTCCCAAACACCAA |  |  |  |  |
| 2 | - | Forward: TCGTCGTCGTTTCGGTTAGTT | 65 | Chr9: 87,498,017-87,498,084 | MSP, MethyLight | [27, 45, 51] |
|  |  | Reverse:TCCCTCCGAAACGCTATCG |  |  |  |  |
|  |  | Probe: 6FAM-CGACCATAAACGCCAACGCCG-BHQ1 |  |  |  |  |
| 3 | - | Forward: GGAGAGGGTGGTTACGGTGTT | 122 | Chr9: 87,498,490-87,498,611 | MethyLight | [37] |
|  |  | Reverse: ACCCTCGCCCAAACGATAC |  |  |  |  |
|  |  | Probe: 6FAM-CAACCTACGACAACGATAA-MGB |  |  |  |  |
| 4 | - | Forward: AGGGGATTCGGTAATTCGTAG1+C | 112 | Chr9: 87,497,931-87,498,042 | qMSP | [40] |
|  |  | Reverse: CCGAAAACTAACCGAAACGAC1+G |  |  |  |  |
|  |  | Probe: 6FAM-TCGGCGTTTGGGAGGGATTTGCGTT-BHQ1 |  |  |  |  |
| 5 | - | Forward: YGGAGGATAGTYGGATYTAGTTAA | 139 | Chr9: 87,497,882-87,498,020 | BSP | [54] |
|  |  | Reverse: ACRAAAACACAACTAAAAAATAAATAAAAAAC |  |  |  |  |
| 6 | - | Forward: GGTTGTTTCGGAGTGTGAGGAGG | 108 | Chr9: 87,497,864-87,497,971 | HRM | [58] |
|  |  | Reverse: GCCGACCCCAAACCCTACC |  |  |  |  |
| 7 | M | Forward: CGTTTGTAGGGTTTTTATTGGTC | 161 | Chr9: 87,497,797-87,497,958 | MSP and qMSP | [59] |
|  |  | Reverse: CTACCGCTACGAATTACCGA |  |  |  |  |
|  | U | Forward: TGTTTGTAGGGTTTTTATTGGTTG | 162 |  |  |  |
|  |  | Reverse: CCTACCACTACAAATTACCA |  |  |  |  |
| M: methylated; U: unmethylated; MSP: methylation-specific PCR; BSP: bisulfite sequencing PCR; NGS: next generation sequencing; | | | | | | |

| **Table S2:** Definitions of 18 items in our quality scoring system. |
| --- |
| **Scientific design** |
| 1.Study objective definition: state the study objectives, prespecified hypothesis or study protocol |
| 2. Sample size: state a statistical sample size or power calculation |
| 3. Population source: state health care setting from which patients were recruited |
| 4. Population selection criteria: state inclusion or exclusion criteria |
| 5. Population demographic characteristics: state the population demographic characteristics (e.g., age, age ate primiparity and menopausal status) |
| 6. Diagnosis of patients: state the criteria or guidelines to diagnose the included patients |
| **Biospecimen management** |
| 1. Biospecimen characteristics: state biospecimen type and anatmical site |
| 2. Biospecimen information: state the methods of collection and storage |
| **Methylation detection** |
| 1. Assay method: state the details of assay method used to detect methylation status (MSP, BSP or pyrosequencing, etc) |
| 2. Primer designs: state the primer sequences |
| 3. Primer sets: use the consistent primer set or detect the consistent region in *DAPK1* |
| 4. Quality control: state the method of quality control |
| **Confounder record** |
| 1. Clinical and pathological data: state the clinical and pathological data (such as tumor type, stage and grade) |
| 2. Conventional risk factors: state the conventional risk factors (such as HPV infection, smoking habit) |
| 3. Other biomarkers: state other biomarker relating with disease (such as methylation status of other genes, point mutation and expression level) |
| **Statistical analysis** |
| 1. Univariate estimate: report the effect of methylation status on outcome |
| 2. Multivariate estimate: adjusted for risk factors or other biomarkers |
| 3. Missing data: state the number of patients with missing data and how to deal with it |

| **Table S3:** Comparative analysis of 13 CPG sites in *DAPK1* between SCC and AdC in the TCGA CESC dataset. | | | | | | | |
| --- | --- | --- | --- | --- | --- | --- | --- |
| CPG sites | SCC | |  | AdC | |  | P ^b^ |
|  | N | Genometric mean (95%CI )^a^ |  | N | Genometric mean (95%CI ) ^a^ |  |  |
| cg08719486 | 254 | 0.771 (0.743-0.800) |  | 53 | 0.572 (0.493-0.664) |  | 5.41 × 10^-5^ |
| cg13823120 | 254 | 0.590 (0.559-0.621) |  | 53 | 0.438 (0.368-0.521) |  | 0.005 |
| cg13814950 | 254 | 0.080 (0.068-0.094) |  | 53 | 0.032 (0.028-0.037) |  | 5.99 × 10^-5^ |
| cg22571217 | 254 | 0.098 (0.084-0.113) |  | 53 | 0.039 (0.034-0.045) |  | **2.75 × 10^-9^** |
| cg13932603 | 254 | 0.064 (0.053-0.076) |  | 52 | 0.023 (0.019-0.026) |  | 1.26 × 10^-5^ |
| cg20401521 | 254 | 0.075 (0.061-0.093) |  | 53 | 0.019 (0.015-0.024) |  | **1.72 × 10^-8^** |
| cg08797471 | 254 | 0.141 (0.127-0.157) |  | 53 | 0.067 (0.059-0.076) |  | **4.08 × 10^-9^** |
| cg19734228 | 254 | 0.241 (0.217-0.268) |  | 53 | 0.094 (0.083-0.108) |  | **7.84 × 10^-13^** |
| cg15746719 | 254 | 0.216 (0.182-0.255) |  | 53 | 0.041 (0.031-0.055) |  | **3.80 × 10^-13^** |
| cg14014720 | 254 | 0.423 (0.385-0.465) |  | 53 | 0.165 (0.140-0.196) |  | **3.95 × 10^-13^** |
| cg13527872 | 252 | 0.444 (0.405-0.487) |  | 53 | 0.178 (0.153-0.206) |  | **1.19 × 10^-13^** |
| cg24754277 | 254 | 0.545 (0.513-0.580) |  | 53 | 0.264 (0.227-0.307) |  | **1.12 × 10^-15^** |
| cg13752933 | 252 | 0.451 (0.420-0.485) |  | 52 | 0.199 (0.165-0.240) |  | **7.61 × 10^-14^** |
| ^a^ Methylation variables were expressed as geometric mean (95%CI) due to ln-transformation before analysis.  ^b^ P values were obtained from the Mann-Whitney U test. | | | | | | | |

| **Table S4:** Associations of 13 CPG sites in *DAPK1* with histological grade of CC in the TCGA CESC dataset. | | | | | | | |
| --- | --- | --- | --- | --- | --- | --- | --- |
| CPG sites | Grade 1 + 2 | |  | Grade 3 | |  | P ^b^ |
|  | N | Genometric mean (95%CI )^a^ |  | N | Genometric mean (95%CI ) ^a^ |  |  |
| cg08719486 | 154 | 0.764 (0.727-0.803) |  | 120 | 0.693 (0.641-0.749) |  | 0.104 |
| cg13823120 | 154 | 0.582 (0.544-0.623) |  | 120 | 0.528 (0.480-0.581) |  | 0.283 |
| cg13814950 | 154 | 0.061 (0.051-0.074) |  | 120 | 0.077 (0.061-0.097) |  | 0.401 |
| cg22571217 | 154 | 0.074 (0.062-0.087) |  | 120 | 0.095 (0.077-0.118) |  | 0.060 |
| cg13932603 | 153 | 0.048 (0.039-0.060) |  | 120 | 0.059 (0.045-0.076) |  | 0.533 |
| cg20401521 | 154 | 0.057 (0.044-0.074) |  | 120 | 0.059 (0.043-0.081) |  | 0.792 |
| cg08797471 | 154 | 0.117 (0.103-0.134) |  | 120 | 0.132 (0.112-0.156) |  | 0.182 |
| cg19734228 | 154 | 0.202 (0.176-0.232) |  | 120 | 0.204 (0.173-0.241) |  | 0.848 |
| cg15746719 | 154 | 0.168 (0.134-0.210) |  | 120 | 0.144 (0.110-0.189) |  | 0.380 |
| cg14014720 | 154 | 0.363 (0.319-0.413) |  | 120 | 0.331 (0.283-0.388) |  | 0.551 |
| cg13527872 | 153 | 0.380 (0.335-0.431) |  | 119 | 0.312 (0.179-0.417) |  | 0.591 |
| cg24754277 | 154 | 0.479 (0.436-0.526) |  | 120 | 0.458 (0.412-0.509) |  | 0.500 |
| cg13752933 | 154 | 0.401 (0.361-0.444) |  | 120 | 0.359 (0.316-0.408) |  | 0.255 |
| ^a^ Methylation variables were expressed as geometric mean (95%CI) due to ln-transformation before analysis.  ^b^ P values were obtained from the Mann-Whitney U test. | | | | | | | |

| **Table S5:** Associations of 13 CPG sites in *DAPK1* with DPF and OS of CC in the TCGA CESC dataset. | | | | | | | | | | | | | | |
| --- | --- | --- | --- | --- | --- | --- | --- | --- | --- | --- | --- | --- | --- | --- |
| CPG sites | Median β value^a^ | OS analysis ^a^ | | | | | |  | DFS analysis ^a^ | | | | | |
|  |  | Without adjustment | | |  | With adjustment | |  | Without adjustment | | |  | With adjustment | |
|  |  | N | HR (95%CI) | P |  | HR (95%CI) | P |  | N | HR (95%CI) | P |  | HR (95%CI) | P |
| cg08719486 | 0.830 | 307 | 0.76 (0.48-1.21) | 0.245 |  | 0.61 (0.31-1.19) | 0.147 |  | 266 | 0.54 (0.30-0.97) | 0.538 |  | 0.61 (0.31-1.19) | 0.147 |
| cg13823120 | 0.640 | 307 | 0.75 (0.47-1.20) | 0.232 |  | 0.63 (0.32-1.22) | 0.170 |  | 266 | 0.57 (0.32-1.03) | 0.063 |  | 0.63 (0.32-1.22) | 0.170 |
| cg13814950 | 0.033 | 307 | 1.06 (0.66-1.69) | 0.806 |  | 1.67 (0.86-3.26) | 0.133 |  | 266 | 1.40 (0.79-2.48) | 0.255 |  | 1.67 (0.86-3.26) | 0.133 |
| cg22571217 | 0.044 | 307 | 0.86 (0.54-1.38) | 0.534 |  | 1.14 (0.57-2.26) | 0.718 |  | 266 | 1.11 (0.62-1.96) | 0.734 |  | 1.14 (0.57-2.26) | 0.718 |
| cg13932603 | 0.023 | 306 | 1.07 (0.67-1.72) | 0.778 |  | 1.24 (0.63-2.41) | 0.536 |  | 266 | 1.30 (0.74-2.31) | 0.365 |  | 1.24 (0.63-2.41) | 0.536 |
| cg20401521 | 0.025 | 307 | 0.92 (0.58-1.47) | 0.725 |  | 0.76 (0.38-1.52) | 0.444 |  | 266 | 0.78 (0.43-1.41) | 0.411 |  | 0.76 (0.38-1.52) | 0.444 |
| cg08797471 | 0.090 | 307 | 0.72 (0.45-1.15) | 0.167 |  | 1.22 (0.61-2.44) | 0.576 |  | 266 | 1.09 (0.61-1.94) | 0.767 |  | 1.22 (0.61-2.44) | 0.576 |
| cg19734228 | 0.167 | 307 | 1.09 (0.69-1.74) | 0.709 |  | 1.01 (0.49-2.07) | 0.975 |  | 266 | 0.86 (0.48-1.52) | 0.599 |  | 1.01 (0.49-2.07) | 0.975 |
| cg15746719 | 0.371 | 307 | 0.98 (0.61-1.56) | 0.927 |  | 0.68 (0.33-1.43) | 0.311 |  | 266 | 0.63 (0.35-1.15) | 0.132 |  | 0.68 (0.33-1.43) | 0.311 |
| cg14014720 | 0.510 | 307 | 1.09 (0.69-1.75) | 0.707 |  | 1.12 (0.54-2.32) | 0.766 |  | 266 | 0.75 (0.42-1.33) | 0.318 |  | 1.12 (0.54-2.32) | 0.766 |
| cg13527872 | 0.512 | 305 | 1.15 (0.72-1.84) | 0.562 |  | 0.86 (0.40-1.83) | 0.693 |  | 264 | 0.66 (0.36-1.20) | 0.658 |  | 0.86 (0.40-1.83) | 0.693 |
| cg24754277 | 0.610 | 307 | 1.49 (0.93-2.39) | 0.096 |  | 1.47 (0.70-3.06) | 0.309 |  | 266 | 0.96 (0.54-1.70) | 0.884 |  | 1.47 (0.70-3.06) | 0.309 |
| cg13752933 | 0.494 | 307 | 0.90 (0.57-1.44) | 0.668 |  | 0.72 (0.36-1.43) | 0.345 |  | 266 | 0.57 (0.32-1.04) | 0.068 |  | 0.72 (0.36-1.43) | 0.345 |
| OS: overall survival; DFS: disease-free survival; N: number; HR (95CI%): hazard ratio (95% confidence interval).  ^a^ For each CPG site, CC patients were classified into two groups based on the median level of β value, then OS ad DFS analyses of 13CPG sites were assessed by the Cox regression approach before and after adjusting for age, FIGO stage, histological grade, and histological type. | | | | | | | | | | | | | | |

| **Table S6:** Effects of 13 CPG sites in *DAPK1* on FIGO stage of CC in a pooled analysis of three TCGA and GEO datasets. | | | | | | | | |
| --- | --- | --- | --- | --- | --- | --- | --- | --- |
| CPG sites ^a^ | Studies N ^a^ | Sample size  (III + IV/ I + II) | Heterogeneity | |  | Effect size | | |
|  |  |  | I^2^(%) | P_Q-test_ |  | Model | SMD (95%CI) | P |
| cg08719486 | 3 | 116/444 | 33 | 0.216 |  | F | -0.03 (-0.23, 0.18) | 0.811 |
| cg13823120 | 2 | 105/408 | 0 | 0.987 |  | F | -0.08 (-0.14, 0.30) | 0.481 |
| cg13814950 | 3 | 116/444 | 0 | 0.725 |  | F | 0.14 (-0.06, 0.35) | 0.173 |
| cg22571217 | 3 | 116/444 | 0 | 0.855 |  | F | 0.15 (-0.06, 0.36) | 0.167 |
| cg13932603 | 2 | 105/407 | 43 | 0.171 |  | F | 0.12 (-0.10, 0.34) | 0.284 |
| cg20401521 | 2 | 54/48 | 0 | 0.661 |  | F | 0.27 (-0.12, 0.66) | 0.182 |
| cg08797471 | 3 | 116/444 | 29 | 0.238 |  | F | 0.10 (-0.10, 0.31) | 0.325 |
| cg19734228 | 3 | 116/444 | 32 | 0.220 |  | F | 0.18 (-0.03, 0.39) | 0.086 |
| **cg15746719** | 3 | 116/444 | 0 | 0.840 |  | F | **0.36 (0.15, 0.57)** | **8.53 × 10^-5^** |
| **cg14014720** | 2 | 105/408 | 0 | 0.803 |  | F | **0.24 (0.03, 0.46)** | **0.029** |
| **cg13527872** | 2 | 105/406 | 0 | 0.864 |  | F | **0.22 (0.00, 0.44)** | **0.047** |
| **cg24754277** | 3 | 116/444 | 0 | 0.695 |  | F | **0.24 (0.03, 0.45)** | **0.023** |
| cg13752933 | 2 | 105/408 | 0 | 0.689 |  | F | 0.16 (-0.06, 0.38) | 0.150 |
| N: number; SMD: standardized mean differences; F: fixed-effects model.  ^a^ TCGA CESC and GSE68339 datasets used the Illumina 450K BeadChip, which included methylation probes of all 13 CPG sites in *DAPK1* promoter; the GSE30760 dataset used 27K BeadChip, which detected seven CPG sites, including cg08719486, cg13814950, cg22571217, cg08797471, cg19734228, cg15746719, and cg24754277.  Bold values indicate significant results with P < 0.05. | | | | | | | | |

| **Table S7:** Associations of 13 CPG sites in *DAPK1* with HSIL risk in a pooled analysis of five GEO datasets. | | | | | | | | |
| --- | --- | --- | --- | --- | --- | --- | --- | --- |
| CPG sites ^a^ | Studies N ^a^ | Sample size  (HSIL/controls) | Heterogeneity | |  | Effect size | | |
|  |  |  | I^2^(%) | P_Q-test_ |  | Model ^b^ | SMD (95%CI) | P |
| cg08719486 | 5 | 106/105 | 22 | 0.276 |  | F | 0.10 (-0.17, 0.38) | 0.469 |
| cg13823120 | 2 | 54/48 | 54 | 0.142 |  | R | -0.06 (-0.66, 0.54) | 0.851 |
| **cg13814950** | 5 | 106/105 | 0 | 0.516 |  | **F** | **0.31 (0.03, 0.58)** | **0.030** |
| cg22571217 | 5 | 106/105 | 18 | 0.301 |  | F | 0.15 (-0.12, 0.43) | 0.279 |
| cg13932603 | 2 | 54/48 | 0 | 0.563 |  | F | 0.24 (-0.16, 0.63) | 0.244 |
| cg20401521 | 2 | 54/48 | 0 | 0.661 |  | F | 0.27 (-0.12, 0.66) | 0.182 |
| **cg08797471** | 5 | 106/105 | 0 | 0.652 |  | **F** | **0.43 (0.15, 0.71)** | **0.003** |
| cg19734228 | 5 | 106/105 | 0 | 0.702 |  | F | 0.21 (-0.07, 0.49) | 0.137 |
| cg15746719 | 5 | 106/105 | 13 | 0.329 |  | F | 0.21 (-0.07, 0.48) | 0.147 |
| cg14014720 | 2 | 54/48 | 82 | 0.023 |  | R | -0.04 (-1.01, 0.92) | 0.926 |
| cg13527872 | 1 | 36/28 | - | - |  | - | 0.17 (-0.33, 0.66) | 0.505 |
| cg24754277 | 5 | 106/105 | 56 | 0.058 |  | R | -0.09 (-0.54, 0.35) | 0.682 |
| cg13752933 | 2 | 54/48 | 64 | 0.086 |  | R | 0.00 (-0.10, 0.11) | 0.953 |
| N: number; SMD: standardized mean differences; F: fixed-effects model; R: random-effects model.  ^a^ TCGA CESC and GSE46306 datasets used the Illumina 450K BeadChip, which included methylation probes of all 13 CPG sites in *DAPK1*; GSE20080, GSE37020, and GSE41384 datasets used 27K BeadChip, which detected seven CPG sites, including cg08719486,cg13814950, cg22571217, cg08797471, cg19734228, cg15746719, and cg24754277.  ^b^ When significant heterogeneity was found (I^2^ ≥ 50% or P_Q-test_ ≤ 0.1), a random-effects model with the inverse variance method was used to pool the results; otherwise, a fixed-effects model was applied.  Bold values indicate significant results with P < 0.05. | | | | | | | | |

| **Table S8:** MeQTL analysis of 13 CPG sites within *DAPK1* promoter in GSE68339 and TCGA CESC datasets. | | | | | | | |
| --- | --- | --- | --- | --- | --- | --- | --- |
| CPG sites | GSE68339 | | |  | TCGA CESC | | |
|  | N | r coefficients ^a^ | P ^a^ |  | N | r coefficients ^a^ | P ^a^ |
| cg08719486 | 121 | -0.254 | 0.005 |  | 309 | -0.329 | 3.26 × 10^-9^ |
| cg13823120 | 121 | -0.277 | 0.002 |  | 309 | -0.233 | 3.51 × 10^-5^ |
| cg13814950 | 121 | -0.448 | 2.65 × 10^-7^ |  | 309 | -0.398 | 3.52 × 10^-13^ |
| cg22571217 | 121 | -0.443 | 3.73 × 10^-7^ |  | 309 | -0.346 | 4.31 × 10^-10^ |
| cg13932603 | 121 | -0.412 | 2.59 × 10^-6^ |  | 308 | -0.391 | 1.03 × 10^-12^ |
| cg20401521 | 121 | -0.340 | 1.34 × 10^-4^ |  | 309 | -0.399 | 3.01 × 10^-13^ |
| cg08797471 | 121 | -0.427 | 1.01 × 10^-6^ |  | 309 | -0.355 | 1.26 × 10^-10^ |
| cg19734228 | 121 | -0.507 | 2.84 × 10^-9^ |  | 309 | -0.547 | 1.55 × 10^-25^ |
| cg15746719 | 121 | -0.421 | 1.49 × 10^-6^ |  | 309 | -0.505 | 2.01 × 10^-21^ |
| cg14014720 | 121 | -0.396 | 6.81 × 10^-6^ |  | 309 | -0.516 | 1.85 × 10^-22^ |
| cg13527872 | 121 | -0.360 | 5.06 × 10^-5^ |  | 307 | -0.517 | 2.11 × 10^-22^ |
| cg24754277 | 121 | -0.342 | 1.25 × 10^-4^ |  | 309 | -0.481 | 2.95 × 10^-19^ |
| cg13752933 | 121 | -0.211 | 0.020 |  | 309 | -0.289 | 2.25 × 10^-7^ |
| ^a^ P values and r coefficients were obtained from the Spearman correlation test. | | | | | | | |

**(B)**

**(A)**

**Figure S1:** Identification of outliers as the major source of heterogeneity by Galbraith plots. (A) Galbraith plots identified two studies by Iliopoulos et al. (No. 21) and Lim et al. (No. 24) as the major contributors of heterogeneity in the comparisons between *DAPK1* promoter hypermethylation and LSL risk; (B) Galbraith plots identified studies by Yang et al. (No. 25), Gasperov et al. (No. 32), and Sun et al. (No. 34) as three contributors of heterogeneity in the comparisons between *DAPK1* promoter hypermethylation and CC risk. Each number is the number of the respective study shown in Table 1.


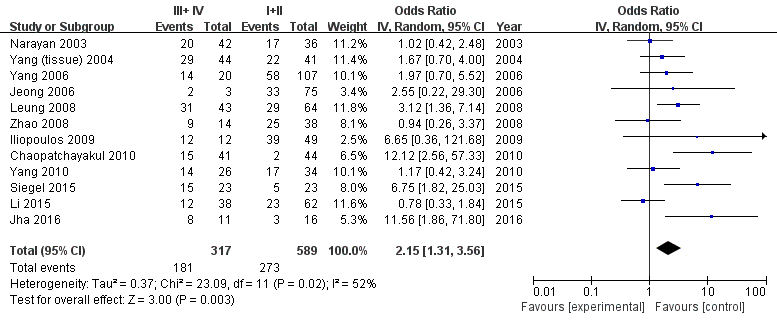


**Figure S3:** Association of *DAPK1* promoter hypermethylation with FIGO stage of CC (III + IV vs I + II) in a meta-analysis of published studies.


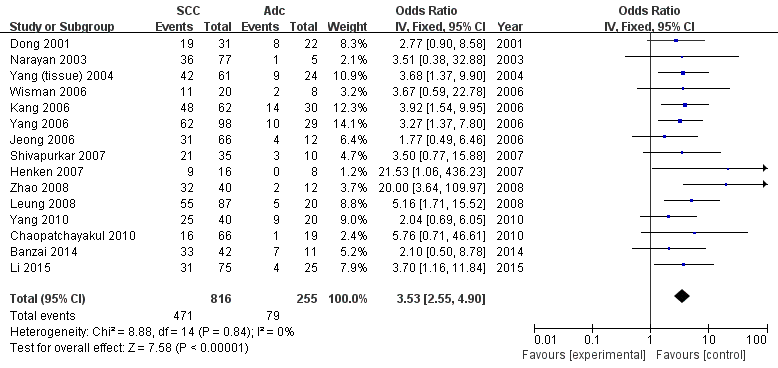


**Figure S2:** Association of *DAPK1* promoter hypermethylation with histological type of CC (SCC vs AdC) in a meta-analysis of published studies.

**(D)**

**(E)**

**(C)**

**(A)**

**(B)**

**Figure S4:** Sensitivity analyses for the associations of *DAPK1* promoter hypermethylation with LSIL risk (A), HSIL risk (B), CC risk (C), histological type of CC (D), and FIGO stage of CC (E). The results were computed by sequentially omitting each study. Bar represents 95% CI. The center of bars represents the summary effects when omitting corresponding studies.


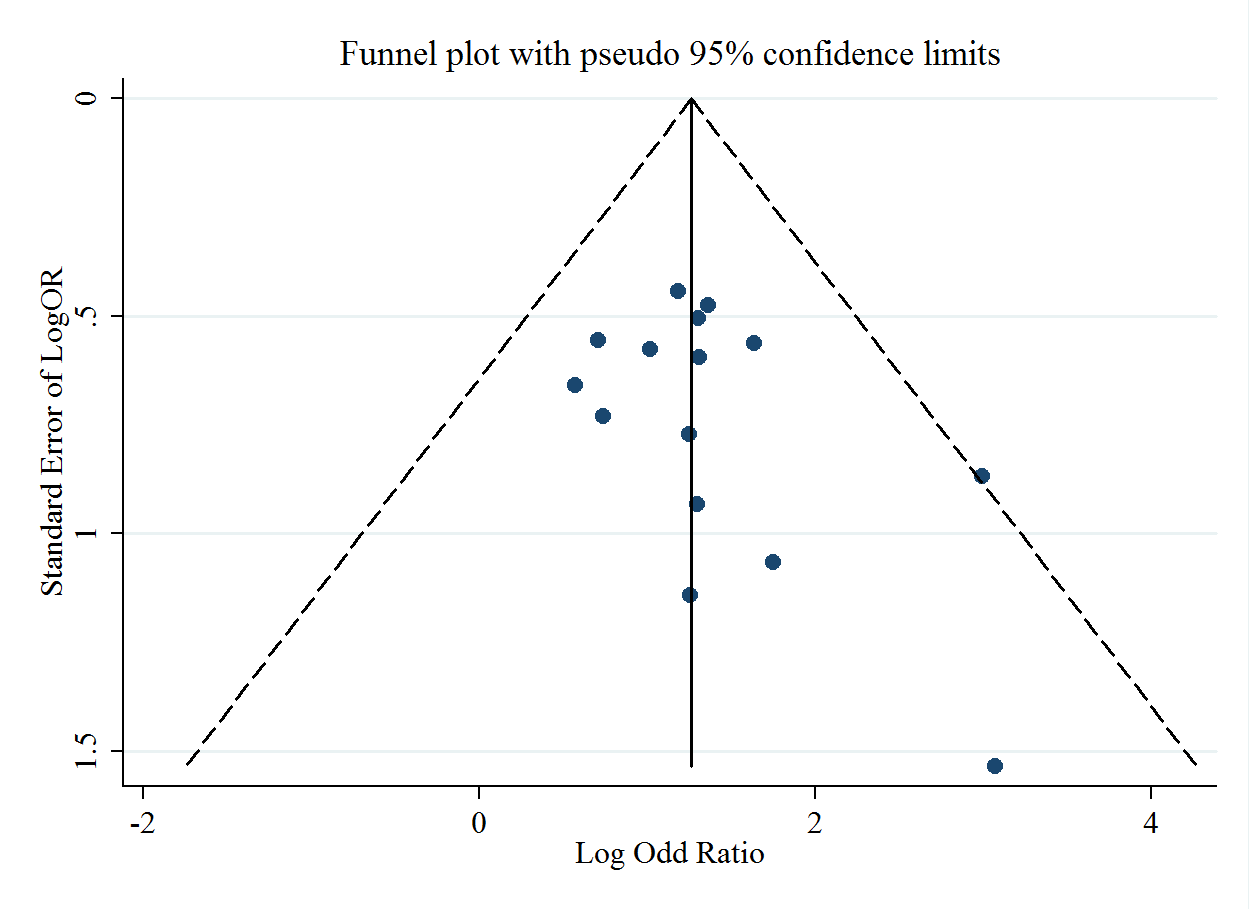


**(D)**


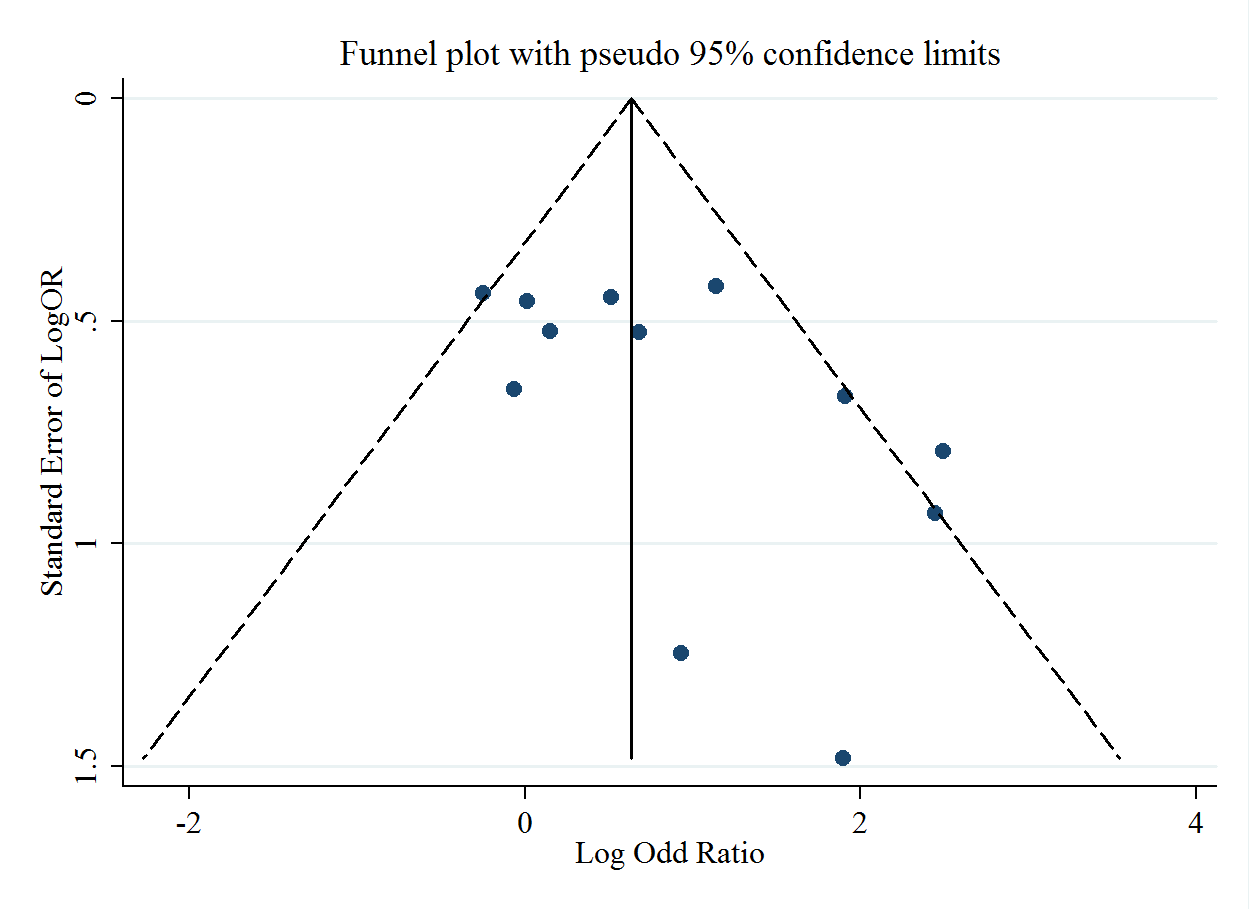


**(E)**


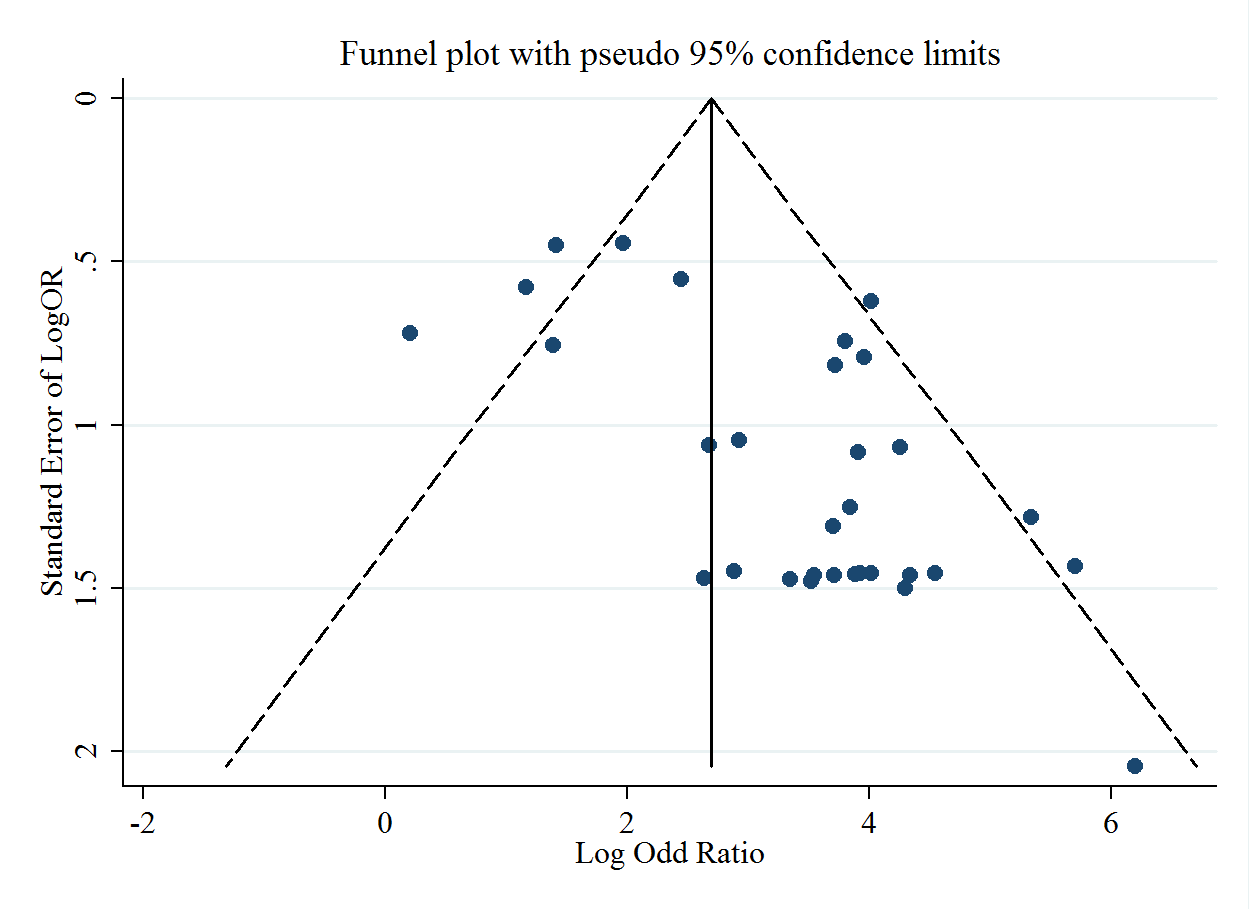


**(C)**


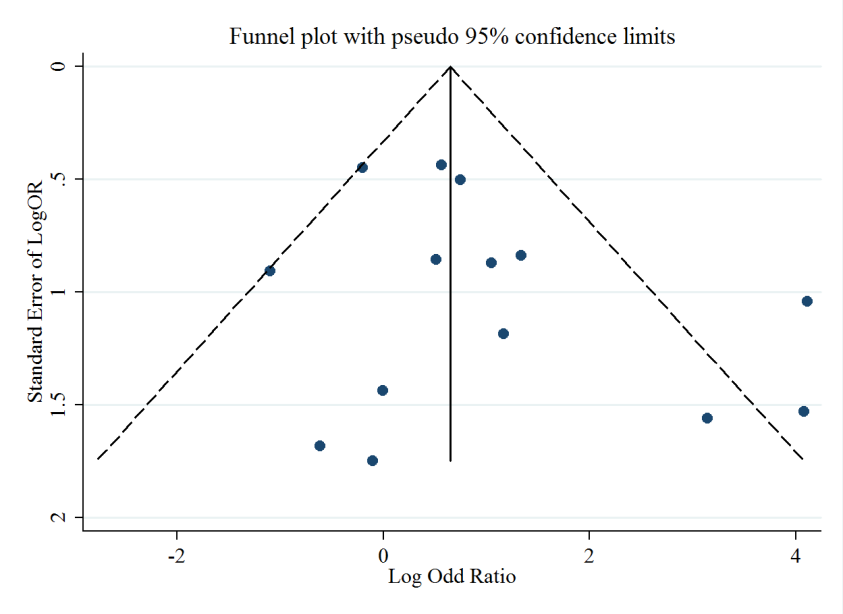


**(A)**


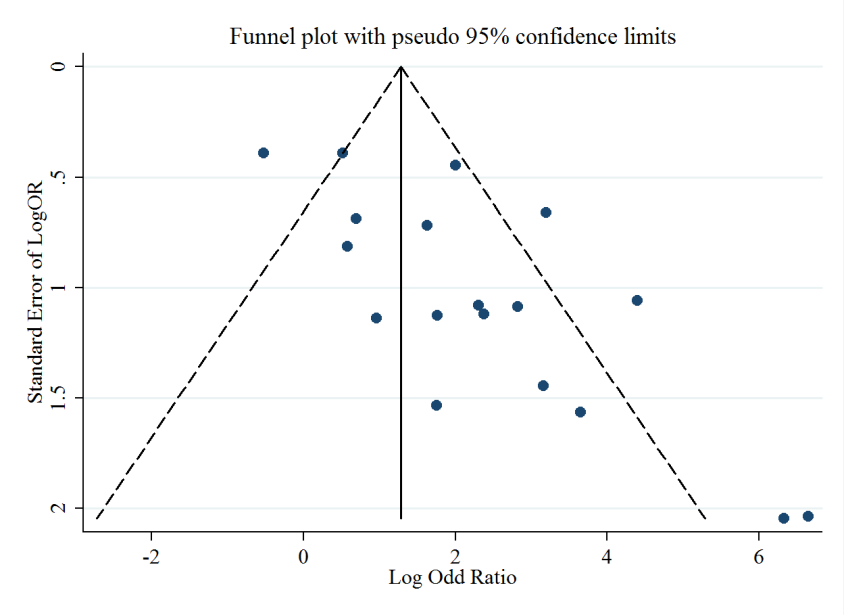


**(B)**

**Figure S5:** Funnel plots for the associations of *DAPK1* promoter hypermethylation with LSIL risk (A), HSIL risk (B), CC risk (C), histological type of CC (D), and FIGO stage of CC (E).


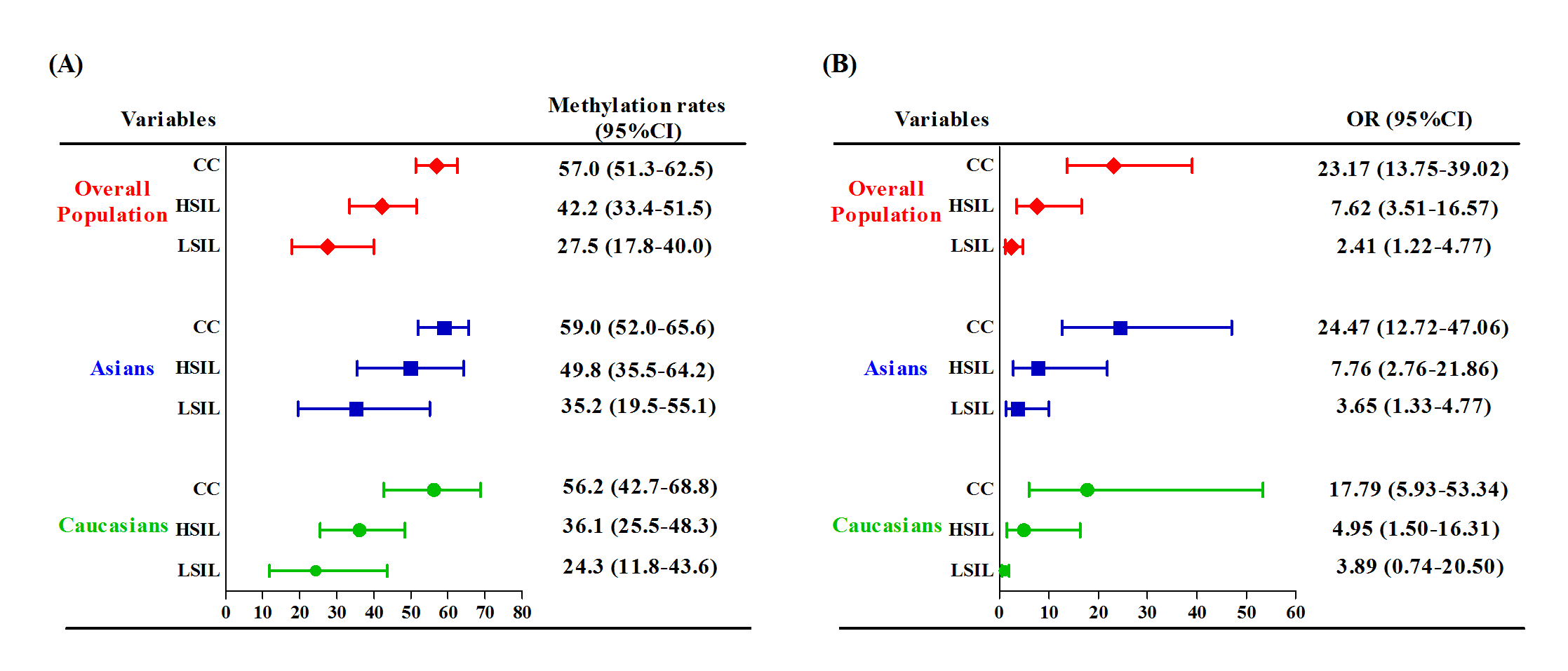


**Figure S6:** Similar effects of *DAPK1* promoter hypermethylation on cervical neoplasia between overall populations, Asians, and Caucasians. (A) Similar hypermethylation rates of *DAPK1* between overall populations, Asians, and Caucasians in cervical neoplasia; (B) Similar effect sizes of *DAPK1* promoter hypermethylation on lesion severity of cervical neoplasia between overall populations, Asians, and Caucasians.
